# When Genome-Based Approach Meets the “Old but Good”: Revealing Genes Involved in the Antibacterial Activity of *Pseudomonas* sp. P482 against Soft Rot Pathogens

**DOI:** 10.3389/fmicb.2016.00782

**Published:** 2016-05-26

**Authors:** Dorota M. Krzyżanowska, Adam Ossowicki, Magdalena Rajewska, Tomasz Maciąg, Magdalena Jabłońska, Michał Obuchowski, Stephan Heeb, Sylwia Jafra

**Affiliations:** ^1^Laboratory of Biological Plant Protection, Department of Biotechnology, Intercollegiate Faculty of Biotechnology of University of Gdansk and Medical University of GdanskGdansk, Poland; ^2^Laboratory of Molecular Bacteriology, Department of Medical Biotechnology, Intercollegiate Faculty of Biotechnology University of Gdansk and Medical University of Gdansk, Medical University of GdanskGdansk, Poland; ^3^School of Life Sciences, Faculty of Medicine and Health Sciences, University of NottinghamNottingham, UK

**Keywords:** *Dickeya*, *Pectobacterium*, secondary metabolites, genome mining, antiSMASH, *nfs*

## Abstract

*Dickeya solani* and *Pectobacterium carotovorum* subsp. *brasiliense* are recently established species of bacterial plant pathogens causing black leg and soft rot of many vegetables and ornamental plants. *Pseudomonas* sp. strain P482 inhibits the growth of these pathogens, a desired trait considering the limited measures to combat these diseases. In this study, we determined the genetic background of the antibacterial activity of P482, and established the phylogenetic position of this strain. *Pseudomonas* sp. P482 was classified as *Pseudomonas donghuensis*. Genome mining revealed that the P482 genome does not contain genes determining the synthesis of known antimicrobials. However, the ClusterFinder algorithm, designed to detect atypical or novel classes of secondary metabolite gene clusters, predicted 18 such clusters in the genome. Screening of a Tn5 mutant library yielded an antimicrobial negative transposon mutant. The transposon insertion was located in a gene encoding an HpcH/HpaI aldolase/citrate lyase family protein. This gene is located in a hypothetical cluster predicted by the ClusterFinder, together with the downstream homologs of four *nfs* genes, that confer production of a non-fluorescent siderophore by *P*. *donghuensis* HYS^T^. Site-directed inactivation of the HpcH/HpaI aldolase gene, the adjacent short chain dehydrogenase gene, as well as a homolog of an essential *nfs* cluster gene, all abolished the antimicrobial activity of the P482, suggesting their involvement in a common biosynthesis pathway. However, none of the mutants showed a decreased siderophore yield, neither was the antimicrobial activity of the wild type P482 compromised by high iron bioavailability. A genomic region comprising the *nfs* cluster and three upstream genes is involved in the antibacterial activity of *P*. *donghuensis* P482 against *D*. *solani* and *P*. *carotovorum* subsp. *brasiliense*. The genes studied are unique to the two known *P*. *donghuensis* strains. This study illustrates that mining of microbial genomes is a powerful approach for predictingthe presence of novel secondary-metabolite encoding genes especially when coupled with transposon mutagenesis.

## Introduction

*Pseudomonas* spp. constitute a highly diverse group of **γ**-proteobacteria, inhabiting various ecological niches (Palleroni, 2005) Over 200 species have been reported in the literature^1^, with at least 144 validated (Gomila et al., 2015). Representatives of the genus include pathogens affecting humans and animals (*Pseudomonas aeruginosa*; Sadikot et al., 2005), insects (*Pseudomonas entomophila*; Vodovar et al., 2005) and plants (mainly *Pseudomonas syringae*; Young, 2010; Baltrus et al., 2011). However, most pseudomonads are harmless commensals, some of which show plant-beneficial or biodegradation properties valuable for biotechnological applications (Adesemoye and Kloepper, 2009; Mercado-Blanco, 2015; Novik et al., 2015). *Pseudomonas* spp. produce a diverse array of biologically active metabolites, including antibiotics, bacteriocins, biosurfactants, toxins, volatiles, and iron-scavenging siderophores (Gross and Loper, 2009; Silby et al., 2011; Schulz-Bohm et al., 2015). Many of these metabolites increase the competitive potential of *Pseudomonas* spp., either by facilitating the colonization of a given niche and nutrient uptake, or through direct influence on the coexisting (micro)organisms (Pliego et al., 2011; Mercado-Blanco, 2015). This competitive edge is of particular importance in environments such as soil and the rhizosphere, where local “hot spots” of microbial activity are formed due to limited and uneven distribution of nutrients.

Numerous *Pseudomonas*-derived metabolites have been studied, many for their antimicrobial activity toward fungi and oomycetes and, to a considerably lesser extent, toward bacteria (Haas and Defago, 2005; Weller, 2007; Gross and Loper, 2009; Pierson and Pierson, 2010; Raaijmakers et al., 2010). Among the antimicrobials, the majority are polyketides (PK), (cyclic)non-ribosomal (lipo)peptides (NRPs or CLPs), or hybrid compounds (PK-NRP; Raaijmakers et al., 2006; Gross and Loper, 2009). Some strains, such as *Pseudomonas protegens* Pf-5 and CHA0^T^ produce several antimicrobial compounds of different chemical classes (Loper et al., 2008; Ramette et al., 2011). The onset of the genomic era has created opportunities for discovering new antimicrobials especially given that over 1800 assemblies of *Pseudomonas* spp. genome sequences are currently available in GenBank (February 2016). The sizes of these genomes range from 4.17 Mbp for *P*. *stutzeri* JM300 (Busquets et al., 2012) to 7.7 Mbp for *P. protegens* Pf-5 (Paulsen et al., 2005). However, the majority of *Pseudomonas* spp. genomes can be considered large (≈6 Mbp), thereby reflecting the pool of genes and regulatory elements necessary to thrive in complex and dynamic environments (Raes et al., 2007; Goldfarb et al., 2011; Silby et al., 2011; Wu et al., 2011; Loper et al., 2012). In addition, the core genome of the genus is relatively small (approximately 25–35%). Taken together, this creates a considerable pool of strain-specific genes, some of which are involved in secondary metabolism conferring unique properties (Loper et al., 2012).

*Pseudomonas* sp. P482 is a tomato rhizosphere isolate, able to inhibit the growth of several plant pathogens, including the stone fruit pathogen *P. syringae* (Golanowska et al., 2012) and various strains of *Dickeya* and *Pectobacterium* (formerly *Erwinia*) genera (Krzyzanowska et al., 2012). The latter are plant pathogens that cause black leg and soft rot diseases of many vegetables and ornamental plants resulting in serious economic losses (Ma et al., 2007). As shown in this study, the spectrum of antibacterial activity of the P482 includes *Dickeya solani* and *Pectobacterium carotovorum* subsp. *brasiliense*, the recently established, highly virulent species of soft rot *Enterobacteriaceae* (SRE; Nabhan et al., 2012; van der Wolf et al., 2014). In the light of limited measures available to protect plants from soft rot (Czajkowski et al., 2011), as well the long history of *Pseudomonas* spp. strains as effective biological control agents against fungal diseases in agriculture and horticulture (Mercado-Blanco, 2015), the discovery of P482 with its novel antibacterial activity is potentially significant. Currently, the only chemically defined *Pseudomonas*-derived compound with antibacterial activity against soft rot bacteria is 2,4-diacetylphloroglucinol (2,4-DAPG). This polyketide antibiotic, although studied mainly for its antifungal properties (i.a., Harrison et al., 1993; Raaijmakers and Weller, 1998) also accounts for the *in vitro* antagonism of *P*. *fluorescens* F113 toward *Erwinia carotovora* subsp. *atroseptica* (currently *Pectobacterium atrosepticum*; Cronin et al., 1997). Despite other reports on *Pseudomonas* strains inhibiting the growth of soft rot bacteria, they all lack information on the mechanism of these antagonistic interactions (Krzyzanowska et al., 2012; Cigna et al., 2015; Raoul des Essarts et al., 2016).

Here, we elucidated the genetic background of the antibacterial activity of *Pseudomonas* sp. P482 toward SRE, with the focus on *D*. *solani* and *P*. *carotovorum* subsp. *brasiliense*. Recent publication of the draft genome of strain P482 (Krzyzanowska et al., 2014), as well as the ongoing development of bioinformatics tools, enabled us to employ genome mining data to identify novel secondary-metabolite gene clusters. Genomic data also enable us to establish the phylogenetic position of P482.

## Materials and methods

### Bacterial strains, culture conditions, and growth rate

Bacterial strains used in this study are listed in Table 1. All strains were cultured in Miller's Lysogeny Broth (LB) or on LB solidified with 1.5% agar (Novagen, Germany). The *Pseudomonas* spp. were grown at 28°C and the *Escherichia coli* ST18 was grown at 37°C. For the growth of the auxotrophic strain *E. coli* ST18, the medium was supplemented with 50 μg·ml^−1^ of 5-aminolevulonic acid (5-ALA; Sigma-Aldrich, USA). When necessary the medium was supplemented with kanamycin (30 μg·ml^−1^). For determination of bacterial growth rate the cells were cultured in 96-well plates and the OD_595_ measurements were performed hourly using an EnVision Multilabel Reader (PerkinElmer, USA).

**Table 1:** **Bacterial strains used in this study**.

**Strain**	**Origin/Features**	**References**
***Pseudomonas*** **spp**.		
*P. aeruginosa* PAO1	Spontaneous chloramphenicol-resistant mutant of the PAO strain, isolated in 1954 from a wound (Australia)	Holloway, 1955, 1975
*P. asplenii* CCM 7744^T^	*Asplenium nidus*; Ark and Tompkins, 1946; Savulescu, 1947 emend. Tvrzová, 2006	Tvrzová et al., 2006
*P. cremoricolorata* DSM 17059^T^	*Oryza sativa* (Japan)	Uchino et al., 2001
*P. donghuensis* HYS^T^	Water sample from the Donghu lake (China)	Gao et al., 2012
*P. entomophila* L48^T^	*Drosophila melanogaster*	Mulet et al., 2012
*P. monteilii* NBRC 103158^T^	Clinical specimen	Elomari et al., 1997
*P. protegens* CHA0^T^	Soil suppressing black root rot of tobacco (*Nicotiana glutinosa*; Switzerland)	Stutz, 1986
*P. protegens* Pf-5	Rhizosphere of cotton (USA)	Howell and Stipanovic, 1979
*P. putida* DSM 291^T^	Type strain; Trevisan, 1889; Migula, 1895	Palleroni, 2005
*P. putida* KT 2440	Soil (Japan), a derivative of mt-2 strain lacking the TOL plasmid	Bagdasarian et al., 1981
*P. vranovensis* DSM 16006^T^	Soil next to a highway (Czech Republic)	Tvrzová et al., 2006
*Pseudomonas* sp. P482	Tomato rhizosphere (Poland)	Krzyzanowska et al., 2012
**SOFT ROT PLANT PATHOGENS**		
*Dickeya solani* IFB 0102	Potato plant (Poland)	Sławiak et al., 2009
*Dickeya solani* IPO 2222^T^	Potato plant (The Netherlands)	van der Wolf et al., 2014
*P. carotovorum* subsp. *brasiliense* LMG21371^T^	Potato plant (Brazil)	Nabhan et al., 2012
*P. carotovorum* subsp. *brasiliense* JJ 56	Potato plant (South Africa)	Thanks to the courtesy of Dr. Jacquie van der Waals (University of Pretoria)
**GENETICALLY MODIFIED STRAINS**		
*Escherichia coli* ST18	Donor strain for diparental mating; *pro thi hsdR*^+^ Tp^R^ Sm^R^; chromosome::RP4-2 Tc::Mu-Kan::Tn7/λpir Δ*hemA*	Thoma and Schobert, 2009
S0405	*Pseudomonas* sp. P482 transposon mutant BV82_4706::mini-Tn5	This study
KN1009, KN3755, KN4705, KN4706, KN4709	*Pseudomonas* sp. P482 mutants carrying an inbuilt pKNOCK-Km suicide vector in the respective loci (BV82_1009–BV82_4709::pKNOCK-Km)	This study

### Phylogenetic analysis based on the 16S rRNA gene, MLSA, and whole-genome ANI

The 16S rRNA gene analysis was performed for partial (1384 nucl.) *rss* gene sequences of 52 *Pseudomonas* species (Table S1). The Multilocus Sequence Analysis (MLSA), performed according to Ramette et al. (2011), involved a concatenated set of partial sequences of three housekeeping genes: *gyrB, rpoB*, and *rpoD*. The total length of the concatenated set was 8328 nucleotides (2415, 4073, and 1840, respectively). All sequence alignments were performed using Clustal Omega (Sievers et al., 2011). Phylogenetic trees were constructed using MEGA 6.06 software (Tamura et al., 2013), Maximum Likelihood method, Kimura two-parameter model with bootstrap support 1000 replicates. Whole-genome average nucleotide identity (ANI) based on BLAST (version 2.2.18) was computed for pairwise alignment of stretches of genomes of using the JSpecies software with default settings^2^ (Richter and Rosselló-Móra, 2009). Apart from the type strains of respective *Pseudomonas* species, the well-studied strains *Pseudomonas putida* KT2440 and *P. protegens* Pf-5 were also subjected to ANI calculations. All nucleotide sequences used for the phylogenetic study were obtained from GenBank^3^, with the exception of the draft genome of *Pseudomonas asplenii* (unpublished data). The accession numbers of the analyzed genes and genomes are provided in Tables S1, S2, respectively.

### Antibacterial activity assay

All bacterial strains for the assay were cultured overnight in LB medium at 28°C. Bacterial cells were harvested by centrifugation and re-suspended in sterile saline (0.9% NaCl). For the soft rot pathogens, the turbidity of each bacterial suspension was adjusted to 1 McFarland unit (DENSILAMETER II, Erba Lachema) and a sterile swab, soaked in the suspension, was used to inoculate the surface of an LB agar plate. For the potential antagonists, the turbidity of bacterial suspensions was adjusted to 4 McFarland units. Two microliter aliquots of each antagonist suspension were spotted on the surface of media pre-inoculated with the pathogens. The samples were incubated at 28°C for 16 h. The diameter of each pathogen inhibition zone was measured and the value obtained was normalized to the diameter of the bacterial antagonist colony forming the zone. Each experiment was performed in triplicate (*n* = 3), unless otherwise stated. To assess the role of iron availability in the antibiosis between P482 and the SRE, the LB agar plates were supplemented with filter-sterilized solution of FeSO_4_ to a final concentration of 15 μM.

### Genome mining for secondary metabolite-encoding genes

The annotated genome of strain P482 (JHTS00000000.1; Krzyzanowska et al., 2014) was searched for the presence of genes involved in the production of 26 metabolites (16 antimicrobials, six siderophores, two biosurfactants, and two compounds with unknown function) reported in literature to be produced by *Pseudomonas* spp. (Table S3). The search was performed at the protein sequence level using the local blastp tool (Altschul, 1997; Altschul et al., 2005) incorporated into Manatee^4^. Query coverage and identity values ≥50% were considered the cutoff values for a positive hit.

Additionally, the genome of the P482 strain was analyzed with an automatic pipeline called the “antibiotics and secondary metabolite analysis shell” (antiSMASH), version 2.0^5^ (Medema et al., 2011; Blin et al., 2013). Both default settings (searched against the software's manually curated database) and settings involving the application of ClusterFinder algorithm (search based on Pfam domain probabilities; Cimermancic et al., 2014) have been used. More detailed information on the particular genes/gene products were obtained from the GenBank^6^ and the KEGG^7^ databases (Kanehisa and Goto, 2000).

### Core and variable genome analysis

The core genome and the pool of variable genes were determined using the EDGAR^8^ tool (Blom et al., 2009). The analyzed group comprised of *Pseudomonas* sp. P482 and three other, P482-related strains: *P. donghuensis* HYS^T^ (AJJP00000000), *P*. *entomophila* L48^T^ (CT573326), and *P. putida* KT2440 (AE015451). For the purpose of the analysis, the genomes of HYS^T^, L48^T^ and KT2440 were re-annotated with the IGS Annotation pipeline, to match the annotation previously obtained for P482 (Krzyzanowska et al., 2014).

### Site-directed mutagenesis

Fragments (316–453 bp) of genes to be inactivated were PCR-amplified using the Hot Start II Phusion DNA polymerase (Thermo Scientific). Details of the primer pairs used, annealing temperatures and the expected amplicons lengths are given in Tables S4, S5. The PCR products obtained were each cloned between the XbaI/XhoI restriction sites of the pKNOCK-Km suicide vector (Alexeyev, 1999). The resulting constructs, designated pKN1009, pKN3755, pKN4705, pKN4706, pKN4707, and pKN4709 (Table 2), were introduced into the *E. coli* ST18 donor strain (Thoma and Schobert, 2009) and subsequently transferred to *Pseudomonas* sp. P482 by biparental mating. In brief, cells from 1.5 ml of overnight LB cultures of both the donor and the recipient were washed twice with fresh LB medium and re-suspended in 0.5 ml of LB. The two suspensions were pooled (1:1) and the cells harvested by centrifugation. Bacterial pellets were re-suspended in a droplet (20–30 μl) of LB and spotted onto an LB agar plate. The sample was incubated for 16 h at 37°C. The macro-colony obtained was scratched from the medium and suspended in 1 ml of LB. One hundred microliter aliquots of the suspension and serial dilutions (10^−1^, 10^−2^, 10^−3^) were plated on LB agar supplemented with kanamycin (30 μg·ml^−1^) but lacking 5-ALA, thus preventing the growth of the *E. coli* ST18. The P482 transconjugants obtained were screened for the presence of the pKNOCK-Km insertion with primers F_pKNOCK_backbone and R_pKNOCK_backbone. To confirm that the suicide vector had incorporated into the target loci, genomic DNA of each mutant was used as template in a sequencing reaction with primer F_outof_pKNOCK. The results obtained enabled mapping of the pKNOCK-Km insertion site to the genome of the P482 strain. The sequencing was performed at Oligo.pl (Warsaw, Poland).

**Table 2:** **Vectors used in this study**.

**Name**	**Properties**	**References**
pRL27	4080 bp; Km^R^; vector for random transposon mutagenesis; *oriRK6, oriT, aph, tetAp*-*tnp*	Larsen et al., 2002
pKNOCK-Km	2098 bp; Km^R^; suicide vector for site-directed mutagenesis; inserts within the target genomic sequence *via* single crossing-over event	Alexeyev, 1999
pKN1009	2515 bp; Km^R^; pKNOCK-Km bearing a 417 bp fragment of BV82_1009 (primers F_XbaI_482_1009/ R_XhoI_482_1009) in the XbaI-XhoI cloning site	This study
pKN3755	2551 bp; Km^R^; pKNOCK-Km bearing a 453 bp fragment of BV82_3755 (primers F_XbaI_482_3755_new/ R_XhoI_482_3755_new) in the XbaI-XhoI cloning site	This study
pKN4705	2420 bp; Km^R^; pKNOCK-Km bearing a 322 bp fragment of BV82_4705 (primers F_XbaI_P482_4705/ R_XhoI_P482_4705) in the XbaI-XhoI cloning site	This study
pKN4706	2414 bp; Km^R^; pKNOCK-Km bearing a 316 bp fragment of BV82_4706 (primers F_XbaI_P482_4706_B/ R_XhoI_P482_4706_B) in the XbaI-XhoI cloning site	This study
pKN4707	2410 bp; Km^R^; pKNOCK-Km bearing a 312 bp fragment of BV82_4707 (primers F_XbaI_P482_4707/ R_XhoI_P482_4707) in the XbaI-XhoI cloning site	This study
pKN4709	2515 bp; Km^R^; pKNOCK-Km bearing a 417 bp fragment of BV82_4709 (primers F_XbaI_P482_4709/ R_XhoI_P482_4709) in the XbaI-XhoI cloning site	This study

### Transposon mutagenesis

Plasmid pRL27 bearing the mini-Tn5 transposon (Larsen et al., 2002) was transferred into *E*. *coli* ST18. The resulting strain *E. coli* ST18 [pRL27] was used to deliver pRL27 into P482 by biparental mating, using the same protocol as described above for the site-directed mutagenesis. The P482 mutants obtained were screened for the loss of antimicrobial activity against soft rot bacteria using *D*. *solani* IFB0102. In the case of mutants showing decreased antimicrobial activity, the integration site of the mini-Tn5 transposon was mapped. For this purpose, the genomic DNA of each mutant was used as a template in sequencing reactions with primers tpnRL13–2_F_LONG and tpnRL17–1_R_LONG (Table S4). Both primers anneal near the ends of the transposon and their 3′ ends face outwards. The resulting sequences, obtained at Oligo.pl (Warsaw, Poland), were used as query in a local blastn search against the genome of the P482 strain.

### Detection of siderophore production on CAS agar

Total siderophore production by the *Pseudomonas* spp. strains studied was assessed on CAS blue agar (Schwyn and Neilands, 1987). Bacterial cells were cultured overnight in LB medium, harvested by centrifugation, and re-suspended in sterile saline (0.9% NaCl). The turbidity of the suspensions was adjusted to 4 McFarland units. Two microliters of each suspension were spotted on CAS agar plates, in two technical replicates. The plates were incubated at 28°C for 24 h, and then another 96 h at room temperature (22°C) for the development of orange halos. Following incubation, the diameter of each halo was measured and the value obtained was normalized to the diameter of the bacterial colony. The experiment was performed twice (*n* = 2), with two technical replicates.

### Pyoverdine production in CAA and MKB media

Two types of iron-poor media: CAA (5 g casamino acids, 1.18 g K_2_HPO_4_·3H_2_O, 0.25 g MgSO_4_·7 H_2_O, per liter; Kümmerli and Brown, 2010) and MKB (5 g casamino acids, 2.5 g K_2_HPO_4_, 15 ml glycerol, 2.5 g MgSO_4_·7 H_2_O, per liter, pH 7.2; Yu et al., 2014), were inoculated (1:1000) with overnight cultures of *Pseudomonas* sp. P482, *P. donghuensis* HYS^T^ and the P482 mutants: KN4705, KN4706, KN4707, KN4709, KN1009, KN3755. Strain *Pseudomonas vranovensis* DSM 16006^T^, a non-fluorescent pseudomonad unable to produce pyoverdine, was used as a reference. Two hundred microliter aliquots of the inoculated media were transferred to the wells of a 96-well plate, in four replicates for each strain and medium. The plates were incubated for 48 h at 28°C, without shaking. Following incubation, the pyoverdine fluorescence level (excitation λ = 400 nm, emission λ = 460 nm) was measured, together with the optical density of the cultures (λ = 600 nm; EnVision Multilabel Plate Reader, Perkin Elmer). The level of pyoverdine production per cell is presented as relative fluorescence units (RFU) = fluorescence (400/460)/OD_600_.

### *In silico* screening for promoter and terminator regions

The sequence of interest (contig JHTS01000055.1, range 27,755–36,623) was analyzed for the presence of sigma housekeeping promoter sequence by three different programs: PromoterHunter^9^ (Klucar et al., 2010), Promoter prediction^10^ (Reese, 2001) and BPROM ^11^ (Solovyev and Salamov, 2011). As the PromoterHunter requires a weight matrix for the −10 and −35 sequences, such a matrix was created based on the *P. aeruginosa* promoters (Potvin et al., 2008), available at phiSITE^12^. The ARNold^13^ program was employed for the identification of rho-independent terminators (Lambert et al., 2004).

### Identification of prophages and genomic islands

The PHAST^14^ software (Zhou et al., 2011; was applied to identify prophage sequences within the draft genome sequence of P482. IslandPick 3.0^15^ (Dhillon et al., 2015) was used to screen the genome for the presence of genomic islands. As part of the IslandPick analysis, the contigs comprising the draft genome were aligned using the genome of *P. entomophila* L48^T^ as a reference. L48^T^ was chosen as the most closely related species for which a complete genomic sequence is available.

## Results

### *Pseudomonas* sp. P482 is a new representative of *P. donghuensis*

**Figure 1:**
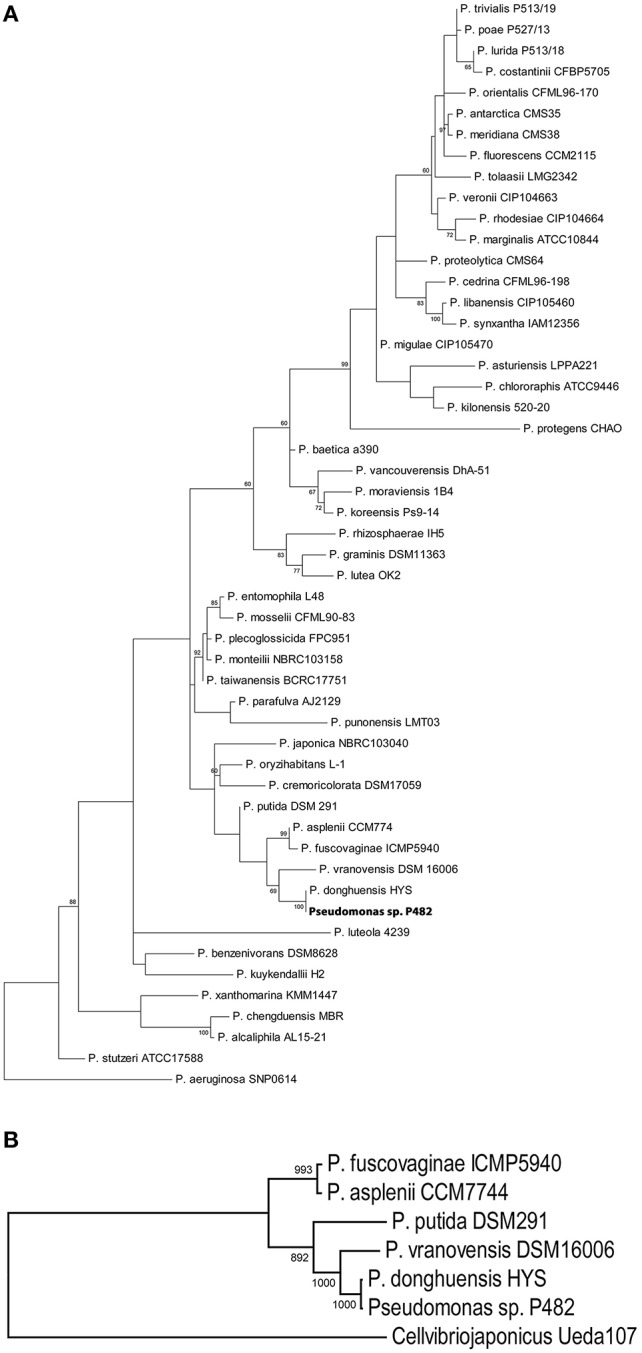
**Phylograms depicting the results of 16S rRNA analysis (A) and MLSA (B) for P482 and related *Pseudomonas* spp. type strains**. The phylogram based on 16S rRNA gene analysis was constructed using maximum likelihood method with Kimura 2-parameter using MEGA 6 software. Bootstrap values are shown at the nodes if the value is >60%. Except for *Pseudomonas* sp. P482, all strains used in the analysis are type strains. *Pseudomonas aeruginosa* SNP 0614 was used as the outgroup. Accession numbers of all the gene sequences included are listed in Table S1. The MLSA was performed for a set of partial nucleotide sequences of three genes: *gyrB, rpoB*, and *rpoD* (8328 nucleotides). *Pseudomonas* sp. P482 and five other *Pseudomonas* spp. strains were included because of the short genetic distance between them (see panel **A**). The MLSA-based phylogram was constructed using maximum likelihood method with GTR + I + G model estimated by jModelTest2 software. Bootstrap values are shown at the nodes. *Cellvibrio japonicas* Ueda107 was used as the outgroup.

**Figure 2:**
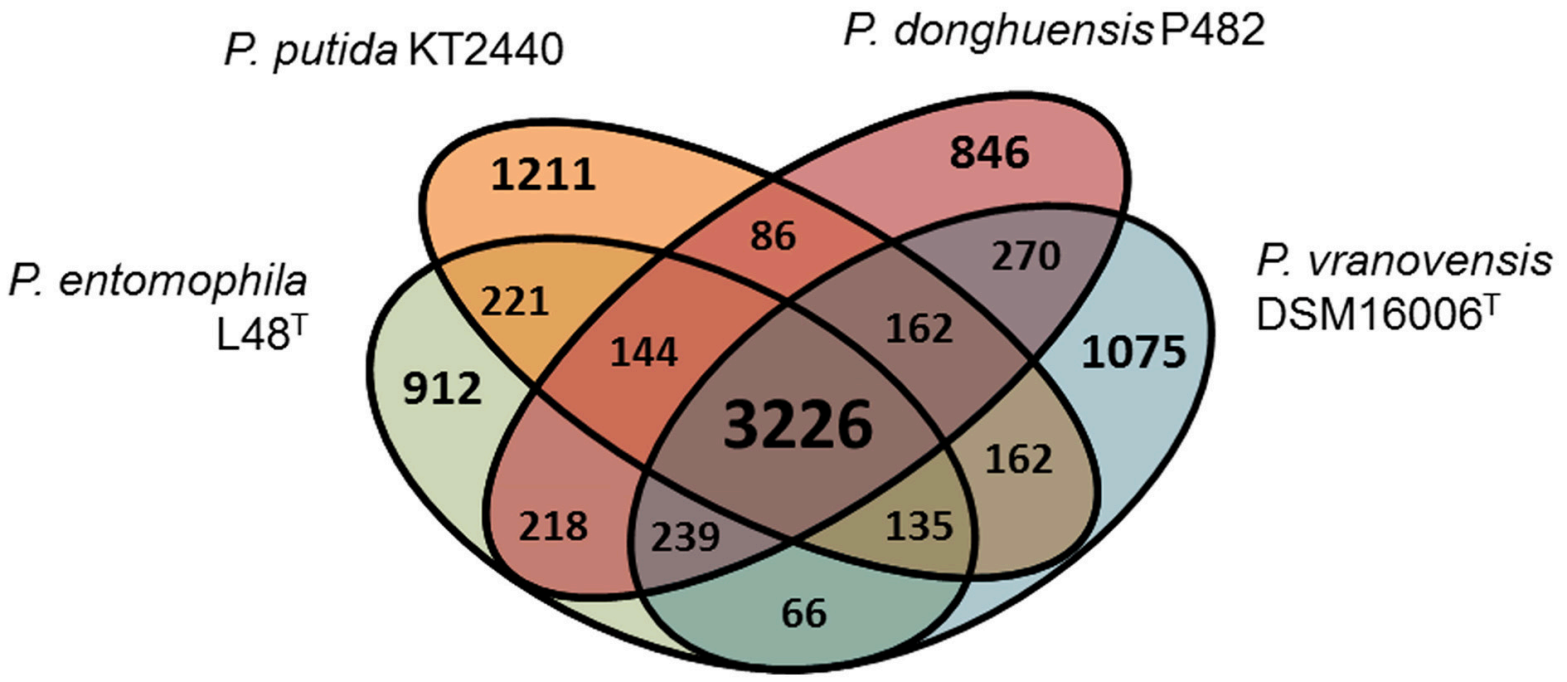
**Venn diagram for the comparison of four *Pseudomonas* spp. genomes, including *P. donghuensis* P482 and three closely related strains**. The calculated core genome (3226 ORFs, shown in large font) represents 60–64% of each genome. The results for *P. donghuensis* HYS^T^ are not shown for clarity. The number of unique ORFs found for the P482 and HYS^T^ was 222 (4.3%) and 345 (6.5%), respectively.

Phylogenetic studies based on the analysis of 16S rRNA gene sequences (Figure 1A) and MLSA (Figure 1B) revealed that the closest relatives of strain P482 are *P. donghuensis* HYS^T^, an isolate from lake water in China studied for high siderophore yield (Gao et al., 2015), *P. vranovensis* DSM 16006^T^, a non-fluorescent pseudomonad obtained from soil next to a highway in Czech Republic (Tvrzová et al., 2006), and a well-studied insect pathogen, *P. entomophila* L48^T^ (Mulet et al., 2012). The availability of genomic data for P482 and the three related strains enabled us to investigate whether P482 belongs to one of these species by calculation of ANI-values for pairwise alignment of genomes (Konstantinidis and Tiedje, 2005; Goris et al., 2007; Richter and Rosselló-Móra, 2009). The highest ANI-value of 99.24% was obtained for the comparison of genomes of strains P482 and HYS^T^ (Table 3). ANI-values calculated for the comparison of genome of P482 with the genomes of *P*. *vranovensis* DSM 16006^T^ and *P. entomophila* L48^T^ were 85.34 and 81.48%. respectively, thus much below the single species value. Considering that the postulated single species threshold is ANI ~ 95–96%, the *Pseudomonas* sp. P482 should be classified as the same species as *P. donghuensis*. This species has recently been established by Gao et al. (2012), with strain HYS^T^ as a type strain and the only known representative. High similarity between P482 and HYS^T^ was also confirmed at the biochemical level using API 20NE and API 50CH (bioMérieux, France; Table S6). A comparative genome analysis using EDGAR showed that the number of unique ORFs found for the P482 and HYS^T^ is 222 (4.3%) and 345 (6.5%), respectively. Some of the differences may have a reason since both genomes are in a draft format. The core genome calculated for P482 and three other related *Pseudomonas* species (*P. vranovensis* DSM 16006^T^, *P*. *entomophila* L48^T^, *P. putida* KT 2440) represents 60–64% of each genome (Figure 2).

**Table 3:** **ANI-values for pairwise alignment of genomes, calculated for the P482 and the type strains of closely related *Pseudomonas* species^a^**.

**Strain**	**ANI (%)^b^**
**SPECIES TYPE STRAINS**	
*Pseudomonas* sp. P482	100
*P. donghuensis* HYS^T^	99.24
*P. vranovensis* DSM 16006^T^	85.34
*P. entomophila* L48^T^	81.48
*P. putida* DSM 291^T^	81.02
*P. monteilii* NBRC 103158^T^	80.78
*P. cremoricolorata* DSM 17059^T^	79.72
*P. protegens* CHA0^T^	79.38
*P. asplenii* CCM 7744^T^	79.09
*P. fuscovaginae* ICMP 5940^T^	78.79
*P. moraviensis* DSM 16007^T^	78.43
**OTHER**	
*P. putida* KT 2440	80.63
*P. ptotegens* Pf-5	79.34

a *The GenBank/EMBL/DDBJ accession numbers for the nucleotide sequences used in this study are listed in Tables S4, S5*.

b *ANI-value (%) for pairwise comparisons of given genomic sequences with the genome of P482. The values given are those obtained for P482 vs. each of the target strains*.

### Comparison of the antibacterial activity of P482 with other related *Pseudomonas* spp.

**Figure 3:**
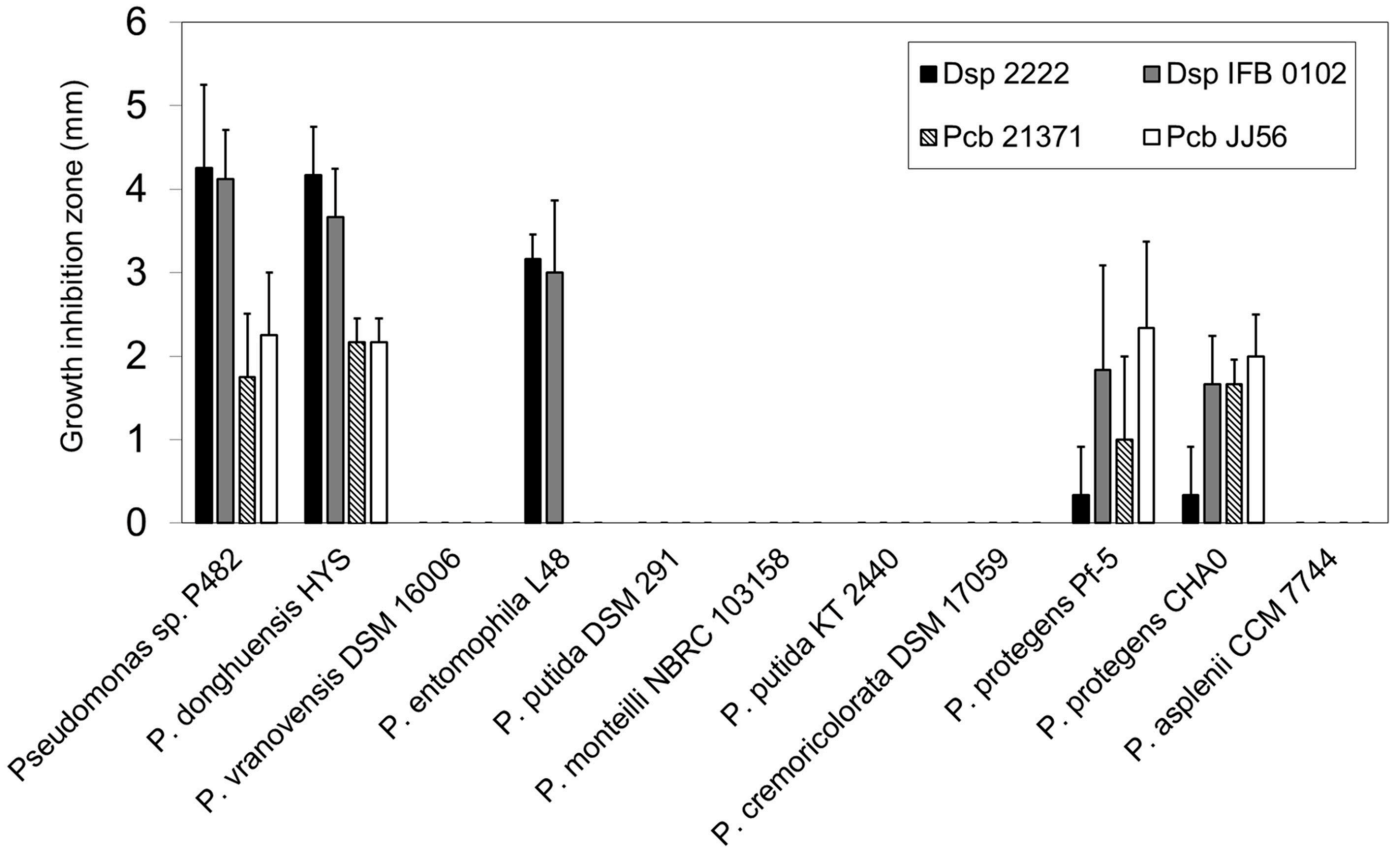
**Antibacterial activity of *Pseudomonas* sp. P482 and the type strains of P482-related species against *D. solani* and *P. carotovorum* subsp. *brasiliense***. Four soft rot bacterial strains were tested: *D. solani* type strain IPO 2222^T^, *D. solani* IFB 0102, *P. carotovorum* subsp. *brasiliense* type strain LMG21371^T^, and *P. carotovorum* subsp. *brasiliense* JJ 56. The order of the *Pseudomonas* strains from left to right reflects the their degree of their relatedness to P482, as estimated from ANI calculations. The histogram shows the mean of three independent experiments, and error bars show standard deviations.

Having established the phylogenetic position of P482, we investigated whether the ability to inhibit *D. solani* and *P*. *carotovorum* subsp. *brasiliense* growth is a unique property of P482, or rather that this trait is more widespread among P482-related species. P482 and 10 *Pseudomonas* spp. strains, closely related to the studied strain based on ANI calculations, were tested for their ability to inhibit the growth of *D. solani* and *P*. *carotovorum* subsp. *brasiliense*. Two strains from each pathogenic species were included in the assay—the type strain (IPO 2222^T^ and LMG21371^T^) and one recently obtained environmental isolate (IFB0102 and JJ 56). This experiment revealed that only *P. donghuensis* HYS^T^ possessed similar antibacterial properties to P482, given that it inhibited growth of all soft rot strains tested (Figure 3). Among other *Pseudomonas* spp. strains, only *P. entomophila* L48^T^ and the two 2,4-DAPG producing *P*. *protegens* strains, CHA0^T^ and Pf-5, showed measurable antibiosis toward the soft rot pathogens. L48^T^ inhibited the growth of *D*. *solani* (approximately 75% activity of that of P482) but was inactive against *P*. *carotovorum* subsp. *brasiliense*. In contrast the *P*. *protegens* strains inhibited the growth of all four pathogens. However, their activity with respect to P482 was relatively high against *P*. *carotovorum* subsp. *brasiliense* (57–100%), but low against *D. solani* (8–44%). This shows that the antimicrobial activity pattern of P482 and HYS^T^ is unique to the two *P*. *donghuensis* strains.

### Mining of the P482 genome for genes conferring the production of biologically active metabolites

#### Manual search focusing on *Pseudomonas*-derived compounds

The genome of P482 was manually searched for the presence of genetic elements essential for the synthesis of biologically active metabolites, previously described for *Pseudomonas* spp. The analysis included 16 metabolites with reported antifungal and/or antibacterial activity (2,4-DAPG, 5-dialkylresorcinols, quinolones, hydrogen cyanide, phenazines, xantholysin, massetolides, mupirocin, orfamides, pyoluteorin, pyrrolnitrin, rhizoxins, syringopeptin, syringomycin and viscosin), six siderophores (achromobactin, quinolobactin/thioquinolobactin, non-fluorescent siderophore, pyoverdin, pseudomonin, pyochelin), two compounds described as biosurfactants (arthrofactin, putisolvin), and two compounds of unknown function (paerucumarin, pseudoverdin). The analysis performed showed that P482 contains genes enabling the production of only three of the compounds investigated: toxic volatile hydrogen cyanide, the siderophore pyoverdine and a chemically undefined, recently described non-fluorescent siderophore of *P*. *donghuensis* HYS^T^ (Yu et al., 2014; Table S3).

#### Automated search using the antiSMASH 2.0 pipeline

The genome of P482 was subjected to an automated search using the “antibiotics and secondary metabolite analysis shell” (antiSMASH 2.0). For detailed results of the antiSMASH analysis see Table 4. When the default settings were applied to the genome of *P*. *donghuensis* P482, five gene clusters were identified: two non-ribosomal peptide synthases (“NRPS”), one designated as “Bacteriocin,” and two classified more generally as “Other.” The two NRPS-type clusters identified consisted of genes potentially involved in the synthesis of pyoverdine (Table 4).

**Table 4:** **Gene clusters potentially involved in the synthesis of secondary metabolites and antibiotics by P482, identified using the antiSMASH 2.0**.

**Cluster^a^**	**Type**	**Scaffold**	**From**	**To**	**Loci/ORFs**	**ORF count**
**Cluster 1**	**Nrps**	**JHTS01000010.1**	**166341**	**219303**	BV82_0986–1026	41
Cluster 2	Putative	JHTS01000014.1	16633	35757	BV82_1430–1442	13
Cluster 3	Putative	JHTS01000015.1	105	16566	BV82_1497–1516	20
Cluster 4	Putative	JHTS01000015.1	45402	54741	BV82_1541–1551	11
Cluster 5	Putative	JHTS01000015.1	210176	219924	BV82_1703–1710	8
Cluster 6	Putative	JHTS01000016.1	152250	159190	BV82_1867–1873	7
**Cluster 7**	**Other**	**JHTS01000023.1**	**2284**	**45037**	**BV82_2006–2035**	**30**
**Cluster 8**	**Other**	**JHTS01000029.1**	**1**	**21146**	**BV82_2407–2421**	**15**
Cluster 9	Putative	JHTS01000032.1	162455	185142	BV82_2572–2597	26
Cluster 10	Putative	JHTS01000032.1	321286	348903	BV82_2717–2740	24
Cluster 11	Putative	JHTS01000032.1	477269	484056	BV82_2856–2661	6
Cluster 12	Putative	JHTS01000032.1	526394	531110	BV82_2896–2900	5
**Cluster 13**	**Bacteriocin**	**JHTS01000037.1**	**1**	**301**	**Non**	**1**
Cluster 14	Putative	JHTS01000040.1	83612	98519	BV82_3374–3388	15
**Cluster 15**	**Nrps**	**JHTS01000045.1**	**70919**	**138413**	**BV82_3735–3785**	**51**
Cluster 16	Putative	JHTS01000048.1	29838	51928	BV82_4156–4173	18
Cluster 17	Putative	JHTS01000048.1	116103	125400	BV82_4236–4245	10
Cluster 18	Putative	JHTS01000055.1	19788	36623	BV82_4697–4712	16
Cluster 19	Putative	JHTS01000062.1	88671	97238	BV82_4995–5004	10
Cluster 20	Putative	JHTS01000065.1	23011	28739	BV82_5054–5059	6
Cluster 21	Putative	JHTS01000065.1	67547	78583	BV82_5091–5100	10
Cluster 22	Putative	JHTS01000065.1	105055	118699	BV82_5122–5131	10
Cluster 23	Putative	JHTS01000067.1	32476	51022	BV82_5172–5192	21

a *Clusters identified using default antiSMASH 2.0 settings are shown in bold and highlighted gray. The remaining putative clusters are the result of an extended antiSMASH 2.0 search, involving the implementation of ClusterFinder algorithm*.

**Figure 4:**
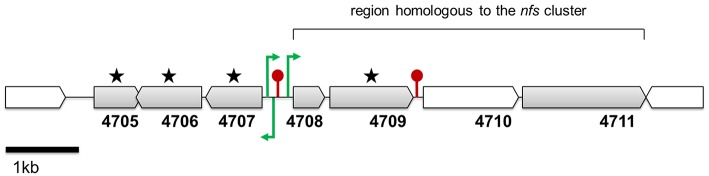
**Genomic region conferring the antibacterial activity of strain *Pseudomonas* sp. P482 toward soft rot bacteria**. Genes marked with stars were inactivated by mutagenesis and the corresponding P482 mutants were impaired in antibacterial activity. ORFs shown in gray encode proteins that have none or few homologs in other *Pseudomonas* spp. The locations of the promoters (green arrows) and the terminators (red pins) are not drawn to scale. Their precise locations in contig JHTS01000055.1 are provided in Supplementary Materials (Tables S9, S10). Annotations of the depicted genes: 4705—bacterial regulatory, tetR family protein; 4706—HpcH/HpaI aldolase/citrate lyase family protein; 4707—short chain dehydrogenase family protein; 4708—thioesterase superfamily protein; 4709—acyl-CoA dehydrogenase, C-terminal domain protein; 4710—phenylacetate-CoA ligase; 4711—thiamine pyrophosphate enzyme.

An “extended” search of the P482 genome, involving the implementation of ClusterFinder, led to the detection of 18 additional gene clusters with the status “Hypothetical” (Table 4). For “Hypothetical” clusters, no suggestions regarding their products and functions are provided. One of the “Hypothetical” clusters, no. 18, incorporated among other, loci BV82_4708–4711 (Figure 4), encoding the homologs of four genes essential for the production of the non-fluorescent siderophore of *P. donghuensis* HYS^T^ (Yu et al., 2014).

Overall this *in silico* search using antiSMASH led to identification of 23 gene clusters, potentially involved in the synthesis of secondary metabolites. None of the clusters was involved in the synthesis of previously described antibiotics of microbial origin. Twenty clusters, comprising 281 genes in total, had no predicted function.

### Identification of genes involved in the antibacterial activity of P482

**Figure 5:**
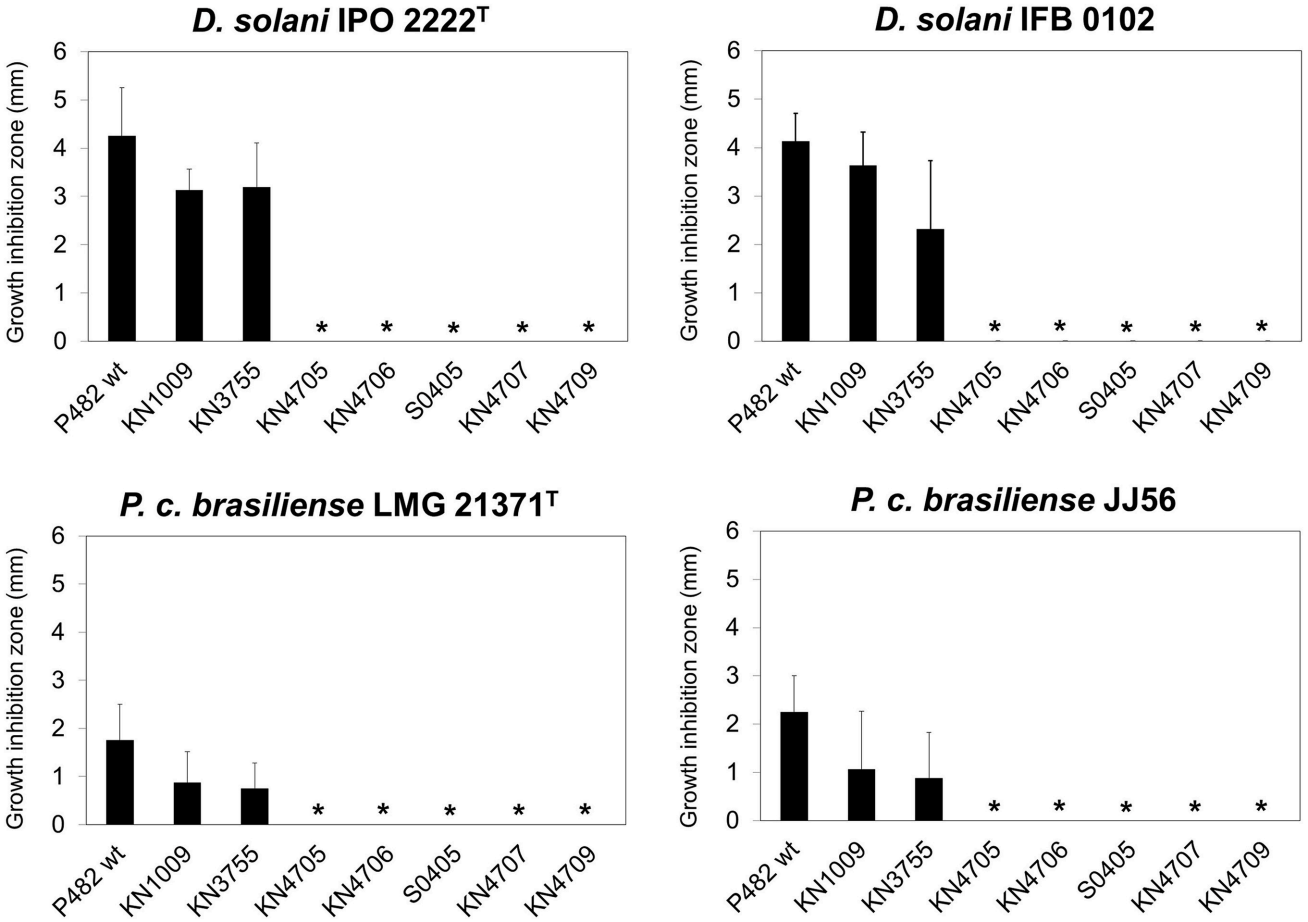
**Antibacterial activity of *Pseudomonas* sp. P482 and its mutants toward *D. solani* and *P. carotovorum* subsp. *brasiliense* strains**. The histogram shows the mean of four independent experiments (*n* = 4), and error bars show standard deviations. Results statistically different from those obtained for the reference strain (P482 wt) in two-tailed Student's *t*-test assuming equal variances (α = 0.05) are marked with stars.

In order to identify gene(s) involved in the antibacterial activity of P482, we performed random mutagenesis using the mini-Tn5 transposon. Screening of over 5000 mutants allowed us to select S0405—a P482 transposon mutant unable to inhibit the growth of *D*. *solani* (IPO 2222^T^, IFB0102) and *P*. *carotovorum* subsp. *brasiliense* (LMG 21371^T^, JJ 56; Figure 5). The transposon insertion in mutant S0405 was located in locus BV82_4706. The product of this gene is annotated as HpcH/HpaI aldolase/citrate lyase family protein, and harbors a domain typical for CitE—the β subunit of the citrate lyase (EC 4.1.3.6). The holoenzyme is involved in the fermentation of citrate by a few bacterial species, and is composed of three protein subunits: α, β and γ, encoded by the *citF, cite*, and *citD* genes, respectively (Bott and Dimroth, 1994; Meyer et al., 1997). The putative CitE from P482 shares only 30% identity to the CitE protein of *Klebsiella pneumoniae* (CAA56216) but is not accompanied by the neighboring *citF* and *citD* genes. In line with this observation, blastp search of the P482 protein dataset using the CitF (CAA56217.1) and CitD (CAA56215.1) sequences as queries gave no relevant matches.

The BV82_4706 gene is located upstream of the homologs of the four genes of the *nfs* cluster from *P*. *donghuensis* HYS^T^ (loci BV82_4708–4711) but oriented in the opposite direction. Moreover, each of the genes described above occurs in one of the antiSMASH-predicted “Hypothetical” clusters, here assigned number 18. At the nucleotide level, the whole of cluster 18 (8522 bp, loci BV82_4697-BV82_4712) shares 99% identity with the region 6854–15376 of Scaffold 1 (AJJP01000005.1) of the draft genome of *P. donghuensis* HYS^T^.

Analysis of cluster 18 in more detail revealed that it consist of 16 open reading frames (loci BV82_4697-BV82_4712; Table 5). Compared with genomic regions found in other *Pseudomonas* spp., cluster 18 can be divided into two parts. The first part (loci BV82_4697-BV82_4704, with the exception of the internally located BV82_4702) encodes proteins having multiple homologs of *Pseudomonas* origin in the NCBI non-redundant protein database (over 100 hits with query coverage >90% and identity >70% each; Table 5). Among these loci, four (BV82_4697-BV82_4700) are putatively involved in the synthesis of alginate—an exopolysaccharide produced by bacteria for protection against environment threats and to enhance adhesion to solid surfaces (Boyd and Chakrabarty, 1995). In contrast, there were few blastp hits of *Pseudomonas* origin for the loci comprising the second part of the cluster (BV82_4705-BV82_4712, with the exception of BV82_4710; 3–20 hits per locus, including the P482 and HYS^T^; Table 5). These results are in line with the comparative genome analysis performed for P482, HYS^T^ and three other related *Pseudomonas* species (*P*. *vranovensis* DSM 16006^T^, *P*. *entomophila* L48^T^, *P*. *putida* KT 2440; Figure 2). This analysis showed that the loci BV82_4705–4711 are among the reading frames that are unique to the *P*. *donghuensis* strains P482 and HYS^T^ (Figure 4).

**Table 5:** **Annotation and features of the genes comprising cluster 18—one of the antiSMASH-predicted gene clusters, putatively responsible for the synthesis of an unknown secondary metabolite(s) by *Pseudomonas* sp. P482**.

**Locus**	**Gene length (bp)**	**Product size (aa)**	**Annotation(s)^a^**	**KEGG [EC]^b^**	**KEGG pathway^c^**	**Number of high score hits (≥ 90% qq, 70% id.) to proteins of taxon *Pseudomonas* spp. (Genbank)^d^^,^^e^**
BV82_4697	1080	359	Alginate lyase (AlgL)	K01729	ko00051	>100
BV82_4698	1458	485	MBOAT, membrane-bound O-acyltransferase family protein	–	–	>100
BV82_4699	1158	385	Putative alginate biosynthesis protein (AlgJ)	–	–	>100
BV82_4700	657	218	Alginate O-acetyl transferase (AlgF) family protein	–	–	>100
BV82_4701	1452	483	Mannose-1-phosphate guanylyltransferase/mannose-6-phosphate isomerase	K16011 [EC:2.7.7.13 5.3.1.8]	ko00051; ko00520	>100
BV82_4702	465	154	Conserved hypothetical protein	–	–	>100
BV82_4703	351	116	Thioredoxin family protein	–	–	10
BV82_4704	825	274	Short chain dehydrogenase family protein	–	–	>100
BV82_4705	642	213	Bacterial regulatory s, tetR family protein	–	–	4
BV82_4706	915	304	HpcH/HpaI aldolase/citrate lyase family protein	–	–	4
BV82_4707	741	246	Short chain dehydrogenase family protein; 3-oxoacyl-[acyl-carrier protein] reductase (KEGG)	K00059 [EC:1.1.1.100]	ko00061; ko00780; ko01040; ko01212	3
BV82_4708	414	137	Thioesterase superfamily protein; acyl-CoA thioester hydrolase (KEGG)	K07107 [EC:3.1.2.-]	–	3
BV82_4709	1143	380	Acyl-CoA dehydrogenase, C-terminal domain protein	–	–	3
BV82_4710	1314	437	Phenylacetate-CoA ligase	K01912 [EC:6.2.1.30]	ko00360	>100
BV82_4711	1692	563	Thiamine pyrophosphate Enzyme, C-terminal; mTPP binding domain protein indolepyruvate decarboxylase (KEGG)	K04103 [EC:4.1.1.74]	ko00380	5
BV82_4712	795	264	Short chain dehydrogenase family protein	–	–	20

a *The genome of P482 was annotated using the IGS annotation engine (Krzyzanowska et al., 2014). If other/more detailed annotations were available for the homologs of the respective gene products (search against the KEGG database), they were given following the IGS annotations*.

b *KEGG annotations obtained using the BlastKOALA tool (http://www.kegg.jp/blastkoala/)*.

c *ko00051—Fructose and mannose metabolism; ko00520—Amino sugar and nucleotide sugar metabolism; ko00061—Fatty acid biosynthesis; ko00780—Biotin metabolism; ko01040—Biosynthesis of unsaturated fatty acids; ko01212—Fatty acid metabolism; ko00360—Phenylalanine metabolism; ko00380—Tryptophan metabolism*.

d *qq—sequence query coverage; id.—sequence identity*.

e *the hits count includes P482, HYS, and the “multispecies” alignment; the analysis was performed in September 2015*.

Based on the results obtained *in silico*, we performed site-directed mutagenesis of four genes, all located in the “unique region” of cluster 18. The loci inactivated were (predicted products in brackets): BV82_4705 (regulatory protein of the TetR family), BV82_4706 (HpcH/HpaI aldolase/citrate lyase family protein), BV82_4707 (short chain acyl dehydrogenase family protein) and BV82_4709 (acyl-CoA dehydrogenase), the latter being an ortholog of the *nfs* cluster essential for non-fluorescent siderophore production (Yu et al., 2014; Figure 4, Table 5). Additionally, we carried out site-directed mutagenesis of loci BV82_1009 (*psvA*/*pvdL*) and BV82_3755 (in part similar to *pvdD* of *P. aeruginosa*), the major genes of the two antiSMASH-predicted “NRPS” clusters, potentially involved in the synthesis of a pyoverdine-like siderophore. Antibacterial activity assays have shown that mutants KN4705, KN4706, KN4707, and KN4709 had completely lost their *in vitro* antagonism toward the soft rot pathogens tested, as observed earlier for the mini-Tn5 mutant S0405 (Figure 5). In contrast, for mutants with disrupted pyoverdine production (KN1009 and KN3755), no statistically significant decrease in the diameter of growth inhibition zones could be observed, with respect to the parental strain P482 (Figure 5). None of the mutants constructed in this study had impaired growth rate in LB medium as used in the antibacterial activity assays (data not shown).

Thus, disruption of the *nfs* cluster, as well as three genes located upstream, affects the production of a compound playing a major role in the *in vitro* antagonism of P482 toward the soft rot bacteria.

### Prediction of promoters and terminators in the genomic region BV82_4705–4712

The cluster 18 region comprising genes BV82_4705–4712 was analyzed for the presence of putative promoters and transcriptional terminators. Three out of several hypothetical promoters were unanimously predicted by all programs used, all located in the spacer region between genes BV82_4707 and BV82_4708 (Figure 4; Table S9). Two of these may drive transcription toward BV82_4708—the first of the four-gene ortholog *nfs* cluster. The third promoter is located in the opposite direction, and may drive transcription toward the BV82_4707 gene. Next, rho-independent terminators were searched using ARNold. Two possible terminator sequences were identified (Table S10). The first terminator is located between loci BV82_4707 and BV82_4708, thus separating the *nfs* cluster homologs from the BV82_4706 and BV82_4707 genes, encoding the HpcH/HpaI aldolase/citrate lyase and the short chain alkyl dehydrogenase, respectively. The second terminator sequence was found between open reading frames BV82_4709 and BV82_4710. The promoters and terminators identified are also present in the HYS^T^ strain. This *in silico* study requires experimental verification. However, both the relative position of the genes and the position of the putative promoters and terminators suggest that the insertional mutagenesis of the BV82_4705, BV82_4706, and BV82_4707 by the pKNOCK system should not affect on the downstream *nfs* loci. Nevertheless, some concerns arise because inactivation of the BV82_4705 gene may influence expression of the partially overlapping BV82_4706.

### Total siderophore production

**Figure 6:**
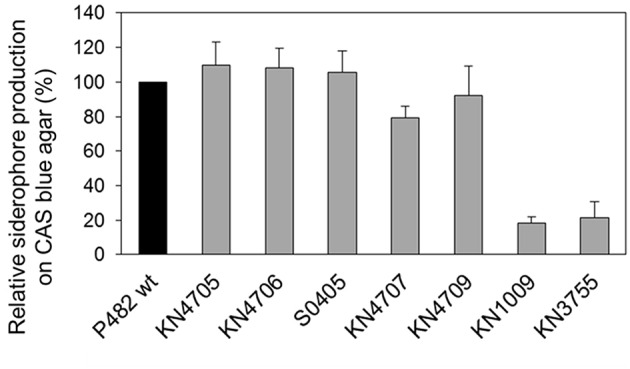
**Total siderophore production on CAS blue agar**. The histogram shows the relative siderophore production (%) by the P482 mutants (gray bars) with respect to the wild type strain (black bar). Error bars indicate standard deviations resulting from two independent experiments.

Total siderophore production on CAS agar was assessed for the P482 wt strain and its mutant derivatives: KN4705, KN4706, S0405, KN4707, KN4709, KN1009, and KN3755. In the case of KN1009 and KN3755, that both contain inactivated pyoverdine synthesis pathway genes, a significant (≈80%) decrease in siderophore production was observed (Figure 6, Figure S1A). The results obtained show that, under these assay conditions, pyoverdine plays the major iron scavenging role, despite the presence of a second siderophore biosynthetic locus. In contrast, the inactivation of the BV82_4709 (*nfs* gene homolog), as well as the three upstream genes (BV82_4705, BV82_4706, and BV82_4707), did not significantly affect total siderophore production (Figure 6).

Additionally, total siderophore production exhibited by P482 was compared with that of HYS^T^ and four other well-studied *Pseudomonas* spp.: *P*. *aeruginosa* PAO1, *P*. *protegens* strains CHA0^T^ and Pf-5, and *P*. *putida* DSM 291^T^. Under the experimental conditions used, strains P482 and HYS^T^ produced comparable levels of siderophores, however, these were markedly lower than CHA0^T^, Pf-5, DSM 291^T^, and PA01 (Figure S1B).

### Production of pyoverdine

**Figure 7:**
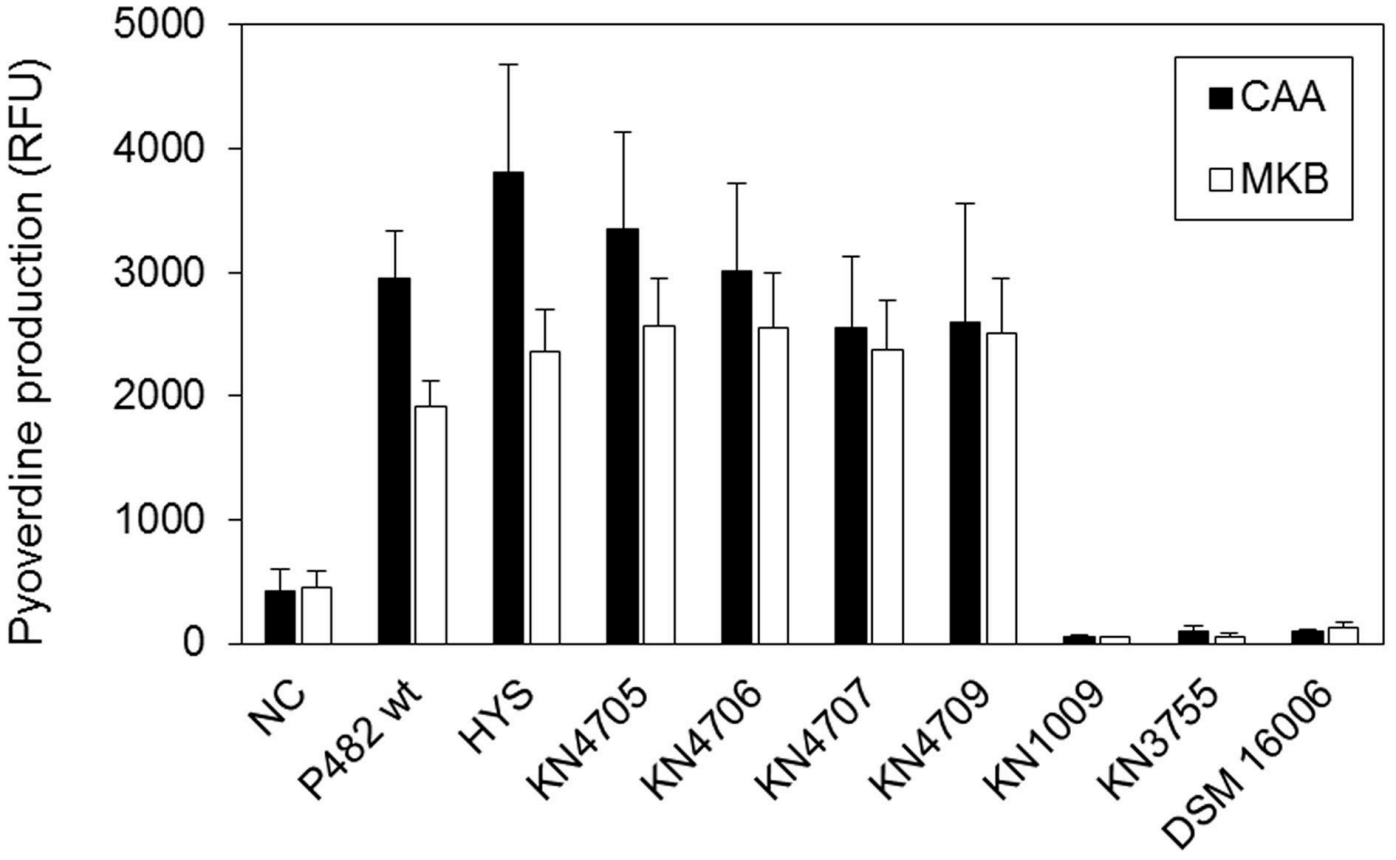
**Pyoverdine production in CAA and MKB media**. The level of pyoverdine production by *Pseudomonas* sp. P482 mutants and the related *Pseudomonas* spp. strains (*P. donghuensis* HYS^T^ and *P. vranovensis* DSM 16006^T^), was measured in two different iron-poor media: CAA (black bars) and MKB (white bars). Results are presented in relative fluorescence units (RFU), calculated as the ratio of fluorescence level (excitation 400 nm, emission 460 nm) to the optical density of the culture at 600 nm. Error bars on the histogram show standard deviations between eight technical replicates from a single experiment.

The *P. donghuensis* strains P482 and HYS^T^ showed comparable levels of pyoverdine in CAA and MKB media (Figure 7). Four P482 mutants KN4705, KN4706, KN4707, and KN4709, showed no significant differences in pyoverdine levels with respect to the wild-type P482. In contrast, the P482 mutants KN1009 and KN3755 in common with *P*. *vranovensis* DSM 16006^T^, do not demonstrate any fluorescent siderophore activity suggesting that they do not produce pyoverdine (Tvrzová et al., 2006). This is consistent with the data mining results which indicated high similarity of the BV82_1009 and BV82_3755 gene products to sequences of proteins involved in pyoverdine synthesis.

### Role of iron availability in the *in vitro* antagonism between P482 and soft rot bacteria

**Figure 8:**
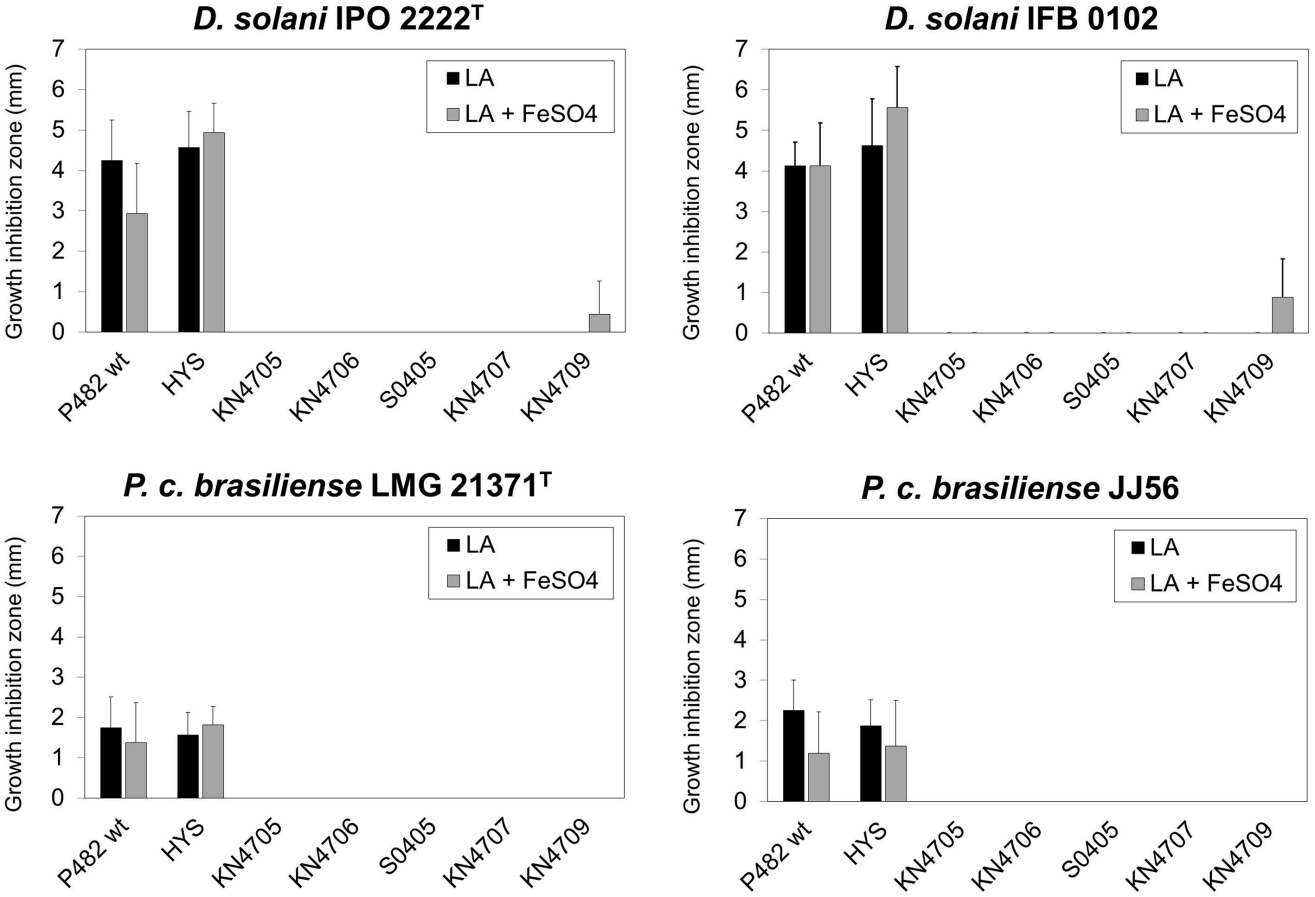
**Influence of iron availability on the antimicrobial activity of *Pseudomonas* sp. P482 and its mutants**. The histogram shows the mean of four independent experiments, and error bars show standard deviations. Black bars represent the diameter of the respective pathogen growth inhibition zone on LB agar medium. The gray bars are the results of the assay performed on LB agar supplemented with 15 μM FeSO_4_.

To assess the influence of high iron availability on the antibacterial activity of P482 toward soft rot bacteria, the growth inhibition assay was performed on LB agar supplemented with 15 μM FeSO_4_. No reduction in growth inhibition zone was observed on this medium for any of the soft rot pathogens (Figure 8). This shows that antagonism between P482 and the soft rot bacteria does not depend on competition for iron alone. Moreover, the antibacterial compound is produced by P482 grown under conditions of high iron availability.

### Investigation of the origin of the genes involved in the antibacterial activity

The P482 draft genome harbors four prophage-like regions, including two prophages scored as “intact” (Table S7), and 11 putative genomic islands (Table S8). Two of the identified genomic islands, consisting of loci BV82_0239—BV82_0241 and BV82_3041—BV82_3048, are directly adjacent to two of the prophage-encoding regions. However, none of the regions showing evidence for horizontal acquisition overlap with the genes of cluster 18 that are involved in the antibacterial activity of P482.

## Discussion

*Pseudomonas* spp. are well-known for their antagonistic properties toward different fungal pathogens as a consequence of the production of antimicrobials and other secondary metabolites (Haas and Defago, 2005; Raaijmakers and Mazzola, 2012). Some of these compounds are also active against bacteria (i.a., Keel et al., 1992), although the mode of antibacterial action of these *Pseudomonas*-derived compounds remains as yet, poorly understood. In this study, we determined the genetic background of the antibacterial activity of strain P482, a fluorescent pseudomonad obtained from the rhizosphere of tomato (Golanowska et al., 2012; Krzyzanowska et al., 2012), toward the pectolytic bacteria *D*. *solani* (van der Wolf et al., 2014) and *P. carotovorum* subsp. *brasiliense* (Nabhan et al., 2012). Over the last decade, these two microorganisms have been reported as highly aggressive plant pathogens causing major economic losses in potato production (Duarte et al., 2004; Sławiak et al., 2009; Tsror et al., 2009; van der Merwe et al., 2009; Laurila et al., 2010; Toth et al., 2011; Leite et al., 2014). Their worldwide dissemination requires the development of novel control strategies, preferably environmentally friendly (i.e., based on natural compounds and microorganisms), as encouraged by European Union regulations (2009/128/EC). Apart from establishing genetic determinants of antibacterial properties of P482, we sought to establish the taxonomic position of this strain.

### *Pseudomonas* sp. P482 is a novel representative of *P. donghuensis*

Our study revealed that P482 should be classified as *Pseudomonas donghuensis*—a species recently established with HYS^T^ as its type strain and sole representative (Gao et al., 2015). The genomes of the P482 and HYS^T^ are estimated to be of comparable size, respectively, 5.639 and 5.648 Mbp (Krzyzanowska et al., 2014; Gao et al., 2015) with an ANI-value for their comparison (the mean identity for the alignments of all homologous genes) exceeding 99%. A comparative genome analysis performed for this pair of strains using EDGAR revealed that the fraction of non-homologous genes, known as the accessory (unique) genome, is only 6.5% (HYS^T^) to 4.3% (P482). Another potential sequence-based relationship between the strains could be the synteny of their genomes, thus the physical co-localization of genetic loci. This, however, cannot be compared for genomes that are still in their draft form, as is the case for both P482 and HYS^T^. Nevertheless, the two strains, P482 and HYS^T^, are highly similar at the genomic level and also phenotypically comparable.

P482 strain was obtained from the rhizosphere of tomato in Gdynia (Poland) and HYS^T^ originates from the waters of Donghu lake (China; Gao et al., 2015). The level of similarity between these microorganisms, isolated at distant locations, suggests a relatively recent common origin. This is likely to be a consequence of spreading *via* the international exchange of plant materials (i.e., Sławiak et al., 2009). However, P482 and HYS^T^ are not the only examples reported of close phylogenetic relations between non-pathogenic *Pseudomonas* spp., isolated from different geographical regions. For example, ANI-values above 98% were calculated for the alignment of the genome of *Pseudomonas* sp. Cab57, obtained from the rhizosphere of a shepherd's purse in Japan, with the genomes of both *P. protegens* CHA0^T^ (98.47%) and *P*. *protegens* Pf-5 (98.22%; Takeuchi et al., 2014). The latter two were isolated from the rhizosphere of tobacco in Switzerland (Stutz, 1986) and the rhizosphere of cotton in USA (Howell and Stipanovic, 1979).

### Growth-inhibitory properties of a novel antimicrobial produced by P482

Multiple secondary metabolites of bacterial origin have been reported, and the genes essential for their synthesis identified (Newman and Cragg, 2012). For our study of *Pseudomonas* sp. P482, we assumed that the growth-inhibitory activity of this strain toward the soft rot pathogens was likely to depend on one or more previously identified compounds. However, mining of the P482 genome failed to reveal any genes involved in the synthesis of known antibiotics. In this respect, P482 was only found to harbor genes enabling the synthesis of hydrogen cyanide and two siderophores: pyoverdine (Cornelis and Matthijs, 2002; Visca et al., 2007) and a non-fluorescent siderophore, previously reported for *P. donghuensis* HYS^T^ (Yu et al., 2014). Hydrogen cyanide, a volatile produced by several bacterial species, is a potent inhibitor of cytochrome c oxidase and several other metalloenzymes, and hence highly toxic for eukaryotic cells (Blumer and Haas, 2000). The headspace volatiles of P482 grown on LB agar did not inhibit the growth of *D. solani* in an *in vitro* assay, thereby excluding contribution of HCN to antibiosis (Ossowicki, unpublished data). The non-ribosomal peptides from the pyoverdine family are high-affinity iron scavengers of the so-called “fluorescent pseudomonads” (Cornelis and Matthijs, 2002; Visca et al., 2007). To our knowledge, these compounds have not been reported as the primary cause of *in vitro* antibiosis between pseudomonads and other bacterial species. In line with this assumption, the pyoverdine-deficient P482 mutants, KN1009 and KN3755, retained their ability to inhibit the growth of the soft rot pathogens.

### Identification of genes involved in the *in vitro* antagonism of P482 toward *D. solani* and *P. carotovorum* subsp. *brasiliense*

Strain S0405, a P482 transposon mutant lacking antibacterial activity against soft rot bacteria, was found to carry the insertion in locus BV82_4706. This gene was annotated as encoding a HpcH/HpaI aldolase family/citrate lyase family protein. Although, the protein contains a domain known from the β subunit (CitE) of the citrate lyase (source: NCBI's CDD, Marchler-Bauer et al., 2014), its similarity to CitE from *K. pneumoniae* is low (Bott and Dimroth, 1994; Meyer et al., 1997). Moreover, P482 lacks genes encoding the α and γ subunits of the CitE holoenzyme, suggesting that the product of BV82_4706 plays a different role in P482 than it does in other citrate-fermenting bacteria (Bekal et al., 1998; Martín et al., 2004). Here, we have also established that the inactivation of the neighboring loci: BV82_4705, BV82_4707, as well as the *nfs* gene homolog BV82_4709, leads to the loss of antibiosis between P482 and the soft rot pathogens.

### Interrelationship between iron availability, siderophore production, and the antimicrobial activity of P482

In *P*. *donghuensis* HYS^T^, the homolog of the BV82_4709 gene was reported to be essential for the synthesis of an, as yet, chemically-undefined non-fluorescent siderophore, responsible for the potent iron-scavenging properties of this strain (Yu et al., 2014). HYS^T^ also produces pyoverdine, however its loss through inactivation of *pvdA* did not reduce the total siderophore yield of HYS^T^, highlighting the compensatory role of the non-fluorescent siderophore. Assuming that these genes play an analogous role in both P482 and HYS^T^, there is overlap between the P482 antibacterial activity, and the siderophore production, reported for HYS^T^. In P482, contrary to the results obtained for HYS^T^, the inactivation of BV82_4709, which corresponds to the *nfs2* ORF of HYS^T^, did not significantly influence total siderophore production by P482. Moreover, two pyoverdine-deficient mutants of the P482, KN1009 (*psvA*^−^), and KN3755 (*pvdD*^−^), showed a profound decrease (approximately 80%) in siderophore production with respect to the wild type strain. Thus, pyoverdine is the major iron chelator employed by P482 in the assay conditions used, even though it is not the only iron-scavenging compound produced by this strain.

Considering the differences between P482 and HYS^T^, it is worth acknowledging that although both groups used CAS agar to detect siderophores, there were technical differences in the experimental setups used. For example, different incubation temperatures were used and it is known that the temperature alters siderophore production levels (Meyer and Stintzi, 1998).

We also investigated the interrelationship between iron availability and the antimicrobial activity of P482. The medium that we routinely use for growth-inhibition assays (LB agar) is relatively iron-rich (~10–17 μM; Goldberg et al., 1990; Abdul-Tehrani et al., 1999). However, additional supplementation with FeSO_4_ can be performed for studies requiring unlimited iron growth conditions (Massé et al., 2005; Ouyang and Isaacson, 2006). Our results showed that the wild type *P*. *donghuensis* P482 inhibits the growth of tested strains of SRE on an iron-rich medium (LB agar supplemented with additional 15 μM of FeSO_4_) in a similar manner to that on unsupplemented LB agar. This implies that the observed *in vitro* antagonism is not based on the competition for iron, and, by extension, on the activity of siderophores. Moreover, the iron-rich conditions do not repress production of antibacterial compound(s) by P482. This is interesting as a FeSO_4_ concentration of 10 μM repressed the production of the non-fluorescent siderophore by *P*. *donghuensis* HYS^T^ (Yu et al., 2014).

Preliminary experiments aiming isolation and purification of the biologically active compound(s) produced by P498, with use the chromatographic and spectrometric techniques failed. Thus, further investigation is required to elucidate the chemical nature of the antibacterial activity of P482 because this cannot be deduced by genomic analysis alone. Similar problem with determination of the chemical problem of novel antimicrobial from *P. putida* W15Oct28 was also described by Ye et al. (2014).

### Antibacterial activity of *P. donghuensis* against soft rot pathogens: unique mechanism of a not so uncommon trait?

The two *P*. *donghuensis* strains, P482 and HYS^T^, exhibit a comparable level of antibiosis toward the *Dickeya* and *Pectobacterium* spp. Among nine related *Pseudomonas* spp. tested, three other strains also hindered the growth of the SRE: the *P. protegens* strains CHA0^T^ and Pf-5, and the *P. entomophila* L48^T^. The CHA0^T^ and Pf-5 produce, among others, 2,4-DAPG, a polyketide that inhibits the growth of *Pectobacterium atrosepticum* (Cronin et al., 1997), *Bacillus cereus, Bacillus thuringensis*, and *Pseudomonas syringae* (MIC-value, 5 μg·ml^−1^), and, to lesser extent, *P. carotovorum* subsp. *carotovorum* (MIC-value, 250 μg·ml^−1^; Keel et al., 1992). To our knowledge, *P. entomophila* L48^T^ has not been previously tested for antibiosis toward the soft rot bacteria. This strain produces many secondary metabolites, including the cyclic lipopeptide xantholysin, shown to have antibacterial activity against xanthomonads and several Gram-positive bacterial species (Li et al., 2013; Molina-Santiago et al., 2015). For CHA0^T^, PF-5 and L48^T^, the spectrum of inhibited soft rot pathogens and/or the size of the growth inhibition zones observed differed from those of P482 and HYS^T^. In general, the antibacterial activity of *P. donghuensis* was more potent than the other strains.

We established that the genes crucial for the production of antimicrobial(s) by P482 are unique to the two known representatives of *P*. *donghuensis*. These genes are absent from the genomes of *P. putida* PA14H7 and *P*. *fluorescens* PA3G8 and PA4C2, three strains previously reported to inhibit the growth of soft rot pathogens (Cigna et al., 2015; Raoul des Essarts et al., 2016). In the latter, *in vitro* screening of over 10000 potato-associated bacterial isolates yielded ~2.4% of strains with antibacterial activity toward at least one soft rot pathogens. Most of the selected antagonistic isolates were classified as *Pseudomonas* or *Bacillus* spp. In earlier work on soft rot antagonists, where we obtained comparable results for rhizobacteria of different herbs and vegetables, approximately 1% of these growth-inhibitors were pseudomonads and bacilli (Krzyzanowska et al., 2012) This provides a rough estimation of how many easily-cultivable bacteria from the sampled environments synthesize, *in vitro*, compounds affecting the growth of soft rot pathogens. This highlights that among *Pseudomonas* spp., this trait is not restricted to a specific strain, species, nor compound. However, for P482 and HYS^T^ genome analysis suggests that they share unique genes, which confer the production of novel antimicrobials.

Genomic regions exclusive to certain *Pseudomonas* spp. strains are often associated with horizontal gene transfer, intragenomic rearrangements, or the activity of mobile genetic elements (Loper et al., 2012). These events are generally recognized as important driving forces of genomic diversity (Darmon and Leach, 2014). However, here we have shown that the BV82_4705–47011 gene cluster of P482, as well as the adjacent genomic regions, do not appear to carry the marks of recent gene transfer (Langille et al., 2008; Zhou et al., 2011).

### Applicability of genome mining for the discovery of novel compounds—the P482 perspective

The development of high-throughput sequencing technologies, as well as user-friendly bioinformatic tools, considerably facilitates the exploration of microbial secondary metabolomes (Donadio et al., 2010; Loman et al., 2012; Blin et al., 2013). Gene/protein-based searches have been effective in linking previously-studied compounds to new isolates, as well as in the discovery of novel compounds synthesized by well-conserved machineries, such as the NRPS, PKS, or terpene synthases (Weber and Marahiel, 2001; Gross et al., 2007; Loper et al., 2008; Rokni-Zadeh et al., 2011; Cane and Ikeda, 2012; Ye et al., 2014; Aleti et al., 2015; Horn et al., 2015; Song et al., 2015). An exciting development is that genome mining tools are now not only able to identify well-known pathways (compounds), but also to provide probability-based guesses of new chemical entities. An automated search of the P482 genome with ClusterFinder, an antiSMASH-integrated algorithm designed to detect atypical or even novel classes of secondary metabolite gene clusters (Cimermancic et al., 2014), led to the prediction of 18 such hypothetical clusters in P482. In total, the number of genes potentially involved in the synthesis of unknown metabolites was estimated to be 281, suggesting a considerable potential for the discovery of diverse novel antimicrobial(s). The value of these predictions is that they enabled discovery of genes essential for the antimicrobial activity of P482. However, this finding could not have been made without combining with transposon mutagenesis, as the high number of hypothetical candidate genes precluded the use of low throughput experimental methods, such as site-directed mutagenesis. Currently, this may be a general limiting factor for the application of genome mining for the discovery of novel secondary metabolites. Overcoming this limitation will be important for the future structural elucidation of compounds encoded by cryptic clusters not active *in vitro* and harbored by both culturable and non-culturable microbes.

## Conclusions

*Pseudomonas* sp. P482 is a novel representative of *Pseudomonas donghuensis* species. The *nfs* cluster and the three upstream genes play a major role in the antibacterial activity of P482 toward the plant pathogens *D. solani* and *P. carotovorum* subsp. *brasiliense*. Moreover, the observed antibiosis does not depend on competition for bioavailable iron. The results obtained so far suggest that the gene cluster identified is unique to the two *P. donghuensis* strains P482 and HYS^T^.

Mining of microbial genomes for genes encoding secondary metabolites is a powerful approach. However, when novel compounds are involved, major limitations are apparent because of the shortage of high-throughput experimental tools to verify the accuracy of *in silico* predictions.

## Author contributions

DK undertook the preliminary research, participated in the design of all experiments, performed genome-mining analyses and ANI calculations, constructed two of the P482 mutants, and wrote the first draft of the manuscript. AO performed the phylogenetic studies, including API biochemical assays, and obtained the P482 transposon mutant. MR performed the siderophore and pyoverdine production assays, contributed to the construction of P482 mutants, prepared growth curves, and critically revised the manuscript. TM constructed three of the mutants reported. MJ performed the antibacterial activity assays. MO performed the searches for promoter and terminator regions and helped to interpret the results. SH contributed to data analysis and revised the manuscript. SJ conceived and co-ordinated the study, helped to plan the experiments and completed the manuscript. All authors approved the final manuscript.

### Conflict of interest statement

The authors declare that the research was conducted in the absence of any commercial or financial relationships that could be construed as a potential conflict of interest.

## Footnotes

^1^www.bacterio.net.

^2^http://www.imedea.uib.es/jspecies.

^3^https://www.ncbi.nlm.nih.gov/genbank/.

^4^http://manatee.sourceforge.net.

^5^http://www.secondarymetabolites.org/.

^6^http://www.ncbi.nlm.nih.gov/.

^7^http://www.genome.jp/kegg/.

^8^http://edgar.computational.bio.uni-giessen.de.

^9^http://www.phisite.org/main/index.php?nav=toolsandnav_sel=hunter.

^10^http://www.fruitfly.org/seq_tools/promoter.html.

^11^http://linux1.softberry.com/berry.phtml?topic=bpromandgroup=programsandsubgroup=gfindb.

^12^http://www.phisite.org/main/index.php?nav=browse.

^13^http://rna.igmors.u-psud.fr/toolbox/arnold/.

^14^http://phast.wishartlab.com/index.html.

^15^http://www.pathogenomics.sfu.ca/islandviewer/.

## References

[B1] Abdul-Tehrani H. Hudson A. J. Chang Y. S. Timms A. R. Hawkins C. Williams J. M.. (1999). Ferritin mutants of *Escherichia coli* are iron deficient and growth impaired, and fur mutants are iron deficient. J. Bacteriol. 181, 1415–1428. 10049371 10.1128/jb.181.5.1415-1428.1999 PMC93529

[B2] Adesemoye A. O. Kloepper J. W. (2009). Plant-microbes interactions in enhanced fertilizer-use efficiency. Appl. Microbiol. Biotechnol. 85, 1–12. 10.1007/s00253-009-2196-0 19707753

[B3] Aleti G. Sessitsch A. Brader G. (2015). Genome mining: prediction of lipopeptides and polyketides from *Bacillus* and related Firmicutes. Comput. Struct. Biotechnol. J. 13, 192–203. 10.1016/j.csbj.2015.03.003 25893081 PMC4397504

[B4] Alexeyev M. F. (1999). The pknock series of broad-host-range mobilizable suicide vectors for gene knockout and targeted DNA insertion into the chromosome of gram-negative bacteria. BioTechniques 26, 824–827. 10337469 10.2144/99265bm05

[B5] Altschul S. (1997). Gapped BLAST and PSI-BLAST: a new generation of protein database search programs. Nucleic Acids Res. 25, 3389–3402. 10.1093/nar/25.17.3389 9254694 PMC146917

[B6] Altschul S. F. Wootton J. C. Gertz E. M. Agarwala R. Morgulis A. Schäffer A. A.. (2005). Protein database searches using compositionally adjusted substitution matrices. FEBS J. 272, 5101–5109. 10.1111/j.1742-4658.2005.04945.x 16218944 PMC1343503

[B7] Ark P. A. Tompkins C. M. (1946). Bacterial leaf blight of bird's-nest fern. Phytopathology 36, 758–761.

[B8] Bagdasarian M. Lurz R. Rückert B. Franklin F. C. Bagdasarian M. M. Frey J. (1981). Specific-purpose plasmid cloning vectors. II. Broad host range, high copy number, RFS 1010-derived vectors, and a host-vector system for gene cloning in *Pseudomonas*. Gene 16, 237–247. 10.1016/0378-1119(81)90080-9 6282695

[B9] Baltrus D. A. Nishimura M. T. Romanchuk A. Chang J. H. Mukhtar M. S. Cherkis K.. (2011). Dynamic evolution of pathogenicity revealed by sequencing and comparative genomics of 19 *Pseudomonas syringae* isolates. PLoS Pathog. 7:e1002132. 10.1371/journal.ppat.1002132 21799664 PMC3136466

[B10] Bekal S. van Beeumen J. Samyn B. Garmyn D. Henini S. Diviès C.. (1998). Purification of *Leuconostoc mesenteroides* citrate lyase and cloning and characterization of the citCDEFG gene cluster. J. Bacteriol. 180, 647–654. 9457870 10.1128/jb.180.3.647-654.1998 PMC106934

[B11] Blin K. Medema M. H. Kazempour D. Fischbach M. A. Breitling R. Takano E.. (2013). Antismash 2.0–A versatile platform for genome mining of secondary metabolite producers. Nucleic Acids Res. 41, W204–W212. 10.1093/nar/gkt449 23737449 PMC3692088

[B12] Blom J. Albaum S. P. Doppmeier D. Pühler A. Vorhölter F.-J. Zakrzewski M.. (2009). Edgar: a software framework for the comparative analysis of prokaryotic genomes. BMC Bioinformatics 10, 154–154. 10.1186/1471-2105-10-154 19457249 PMC2696450

[B13] Blumer C. Haas D. (2000). Mechanism, regulation, and ecological role of bacterial cyanide biosynthesis. Arch. Microbiol. 173, 170–177. 10.1007/s002039900127 10763748

[B14] Bott M. Dimroth P. (1994). *Klebsiella pneumoniae* genes for citrate lyase and citrate lyase ligase: localization, sequencing, and expression. Mol. Microbiol. 14, 347–356. 10.1111/j.1365-2958.1994.tb01295.x 7830578

[B15] Boyd A. Chakrabarty A. M. (1995). *Pseudomonas aeruginosa* biofilms: role of the alginate exopolysaccharide. J. Ind. Microbiol. 15, 162–168. 10.1007/BF01569821 8519473

[B16] Busquets A. Peña A. Gomila M. Bosch R. Nogales B. García-Valdés E.. (2012). Genome sequence of *Pseudomonas stutzeri* strain JM300 (DSM 10701), a soil isolate and model organism for natural transformation. J. Bacteriol. 194, 5477–5478. 10.1128/JB.01257-12 22965097 PMC3457215

[B17] Cane D. E. Ikeda H. (2012). Exploration and mining of the bacterial terpenome. Accounts Chem. Res. 45, 463–472. 10.1021/ar200198d 22039990 PMC3288161

[B18] Cigna J. Raoul Des Essarts Y. Mondy S. Hélias V. Beury-Cirou A. Faure D. (2015). Draft genome sequences of *Pseudomonas fluorescens* strains PA4C2 and PA3G8 and *Pseudomonas putida* PA147, three biocontrol bacteria against *Dickeya* phytopathogens. Genome Announc. 3, e01503-14. 10.1128/genomeA.01503-14 25635023 PMC4319517

[B19] Cimermancic P. Medema M. H. Claesen J. Kurita K. Wieland Brown L. C. Mavrommatis K.. (2014). Insights into secondary metabolism from a global analysis of prokaryotic biosynthetic gene clusters. Cell 158, 412–421. 10.1016/j.cell.2014.06.034 25036635 PMC4123684

[B20] Cornelis P. Matthijs S. (2002). Diversity of siderophore-mediated iron uptake systems in fluorescent pseudomonads: not only pyoverdines. Environ. Microbiol. 4, 787–798. 10.1046/j.1462-2920.2002.00369.x 12534462

[B21] Cronin D. Moënne-Loccoz Y. Fenton A. Dunne C. Dowling D. N. O'gara F. (1997). Ecological interaction of a biocontrol *Pseudomonas fluorescens* strain producing 2,4-diacetylphloroglucinol with the soft rot potato pathogen *Erwinia carotovora* subsp. *atroseptica*. FEMS Microbiol. Ecol. 23, 95–106. 10.1111/j.1574-6941.1997.tb00394.x

[B22] Czajkowski R. Perombelon M. C. M. van Veen J. A. van der Wolf J. M. (2011). Control of blackleg and tuber soft rot of potato caused by *Pectobacterium* and *Dickeya* species: a review. Plant Pathol. 60, 999–1013. 10.1111/j.1365-3059.2011.02470.x

[B23] Darmon E. Leach D. R. F. (2014). Bacterial genome instability. Microbiol. Mol. Biol. Rev. 78, 1–39. 10.1128/MMBR.00035-13 24600039 PMC3957733

[B24] Dhillon B. K. Laird M. R. Shay J. A. Winsor G. L. Lo R. Nizam F.. (2015). Islandviewer 3: more flexible, interactive genomic island discovery, visualization and analysis. Nucleic Acids Res. 43, W104–W108. 10.1093/nar/gkv401 25916842 PMC4489224

[B25] Donadio S. Maffioli S. Monciardini P. Sosio M. Jabes D. (2010). Antibiotic discovery in the twenty-first century: current trends and future perspectives. J. Antibiot. 63, 423–430. 10.1038/ja.2010.62 20551985

[B26] Duarte V. de Boer S. H. Ward L. J. De Oliveira A. M. R. (2004). Characterization of atypical *Erwinia carotovora* strains causing blackleg of potato in brazil. J. Appl. Microbiol. 96, 535–545. 10.1111/j.1365-2672.2004.02173.x 14962133

[B27] Elomari M. Coroler L. Verhille S. Izard D. Leclerc H. (1997). *Pseudomonas monteilii* sp. nov., isolated from clinical specimens. Int. J. Syst. Bacteriol. 47, 846–852. 10.1099/00207713-47-3-846 9226917

[B28] Gao J. Xie G. Peng F. Xie Z. (2015). *Pseudomonas donghuensis* sp. nov., exhibiting high-yields of siderophore. Antonie Van Leeuwenhoek 107, 83–94. 10.1007/s10482-014-0306-1 25331337

[B29] Gao J. Yu X. Xie Z. (2012). Draft genome sequence of high-siderophore-yielding *Pseudomonas* sp. strain HYS. J. Bacteriol. 194, 4121–4121. 10.1128/jb.00688-12 22815441 PMC3416532

[B30] Golanowska M. Ankiewicz H. Taraszkiewicz A. Kamysz W. Czajkowski R. Krolicka A. (2012). Combined effect of the antagonistic potential of selected *Pseudomonas* spp. strains and the synthetic peptide “CAMEL” on *Pseudomonas syringae* pv. *syringae* and *P. syringae* pv. *morsprunorum*. J. Plant. Pathol. 94, S1.69–S61.73. 10.4454/jpp.v94i1sup.012

[B31] Goldberg M. B. Dirita V. J. Calderwood S. B. (1990). Identification of an iron-regulated virulence determinant in *Vibrio cholerae*, using tnphoa mutagenesis. Infect. Immun. 58, 55–60. 2152889 10.1128/iai.58.1.55-60.1990 PMC258408

[B32] Goldfarb K. C. Karaoz U. Hanson C. A. Santee C. A. Bradford M. A. Treseder K. K.. (2011). Differential growth responses of soil bacterial taxa to carbon substrates of varying chemical recalcitrance. Front. Microbiol. 2:94. 10.3389/fmicb.2011.00094 21833332 PMC3153052

[B33] Gomila M. Peña A. Mulet M. Lalucat J. García-Valdés E. (2015). Phylogenomics and systematics in *Pseudomonas*. Front. Microbiol. 6:214 10.3389/fmicb.2015.00214 PMC4447124 26074881

[B34] Goris J. Konstantinidis K. T. Klappenbach J. A. Coenye T. Vandamme P. Tiedje J. M. (2007). DNA–DNA hybridization values and their relationship to whole-genome sequence similarities. Int. J. Syst. Evol. Microbiol. 57, 81–91. 10.1099/ijs.0.64483-0 17220447

[B35] Gross H. Loper J. E. (2009). Genomics of secondary metabolite production by *Pseudomonas* spp. Nat. Prod. Rep. 26, 1408–1446. 10.1039/b817075b 19844639

[B36] Gross H. Stockwell V. O. Henkels M. D. Nowak-Thompson B. Loper J. E. Gerwick W. H. (2007). The genomisotopic approach: a systematic method to isolate products of orphan biosynthetic gene clusters. Chem. Biol. 14, 53–63. 10.1016/j.chembiol.2006.11.007 17254952

[B37] Haas D. Defago G. (2005). Biological control of soil-borne pathogens by fluorescent pseudomonads. Nat. Rev. Microbiol. 3, 307–319. 10.1038/nrmicro1129 15759041

[B38] Harrison L. A. Letendre L. Kovacevich P. Pierson E. Weller D. (1993). Purification of an antibiotic effective against *Gaeumannomyces graminis* var. *tritici* produced by a biocontrol agent, *Pseudomonas aureofaciens*. Soil Biol. Biochem. 25, 215–221. 10.1016/0038-0717(93)90029-B

[B39] Holloway B. (1975). Genetics and Biochemistry of Pseudomonas. London: Wiley and Sons Ltd.

[B40] Holloway B. W. (1955). Genetic recombination in *Pseudomonas aeruginosa*. J. Gen. Microbiol. 13, 572–581. 10.1099/00221287-13-3-572 13278508

[B41] Horn H. Hentschel U. Abdelmohsen U. R. (2015). Mining genomes of three marine sponge-associated actinobacterial isolates for secondary metabolism. Genome Announc. 3:e01106-15. 10.1128/genomeA.01106-15 26430030 PMC4591302

[B42] Howell C. R. Stipanovic R. D. (1979). Control of *Rhizoctonia solani* on cotton seedlings with *Pseudomonas fluorescens* and with an antibiotic produced by the bacterium. Phytopathology 69, 480–482. 10.1094/Phyto-69-480

[B43] Kanehisa M. Goto S. (2000). Kegg: Kyoto encyclopedia of genes and genomes. Nucleic Acids Res. 28, 27–30. 10.1093/nar/28.1.27 10592173 PMC102409

[B44] Keel C. Schnider U. Maurhofer M. Voisard C. Laville J. Burger U. (1992). Suppression of root diseases by *Pseudomonas fluorescens* CHA0: importance of the bacterial secondary metabolite 2,4-diacetylphloroglucinol. Mol. Plant Microbe Interact. 5, 4–13. 10.1094/MPMI-5-004

[B45] Klucar L. Stano M. Hajduk M. (2010). phiSITE: database of gene regulation in bacteriophages. Nucleic Acids Res. 38, D366–D370. 10.1093/nar/gkp911 19900969 PMC2808901

[B46] Konstantinidis K. T. Tiedje J. M. (2005). Genomic insights that advance the species definition for prokaryotes. Proc. Natl. Acad. Sci. U.S.A. 102, 2567–2572. 10.1073/pnas.0409727102 15701695 PMC549018

[B47] Krzyzanowska D. M. Ossowicki A. Jafra S. (2014). Genome sequence of *Pseudomonas* sp. strain P482, a tomato rhizosphere isolate with broad-spectrum antimicrobial activity. Genome Announc. 2:e00394-14. 10.1128/genomea.00394-14 24970823 PMC4073107

[B48] Krzyzanowska D. Potrykus M. Golanowska M. Polonis K. Gwizdek-Wisniewska A. Lojkowska E. (2012). Rhizosphere bacteria as potential biocontrol agents against soft rot caused by various *Pectobacterium* and *Dickeya* spp. strains. J. Plant Pathol. 94, 367–378. 10.4454/JPP.FA.2012.042

[B49] Kümmerli R. Brown S. P. (2010). Molecular and regulatory properties of a public good shape the evolution of cooperation. Proc. Natl. Acad. Sci. U.S.A. 107, 18921–18926. 10.1073/pnas.1011154107 20944065 PMC2973908

[B50] Lambert A. Fontaine J.-F. Legendre M. Leclerc F. Permal E. Major F. (2004). The ERPIN server: an interface to profile-based rna motif identification. Nucleic Acids Res. 32, W160–W165. 10.1093/nar/gkh418 15215371 PMC441556

[B51] Langille M. G. I. Hsiao W. W. L. Brinkman F. S. L. (2008). Evaluation of genomic island predictors using a comparative genomics approach. BMC Bioinformatics 9, 329–329. 10.1186/1471-2105-9-329 18680607 PMC2518932

[B52] Larsen R. A. Wilson M. M. Guss A. M. Metcalf W. W. (2002). Genetic analysis of pigment biosynthesis in *Xanthobacter autotrophicus* Py2 using a new, highly efficient transposon mutagenesis system that is functional in a wide variety of bacteria. Arch. Microbiol. 178, 193–201. 10.1007/s00203-002-0442-2 12189420

[B53] Laurila J. Hannukkala A. Nykyri J. Pasanen M. Hélias V. Garlant L. (2010). Symptoms and yield reduction caused by *Dickeya* spp. strains isolated from potato and river water in Finland. Eur. J. Plant. Pathol. 126, 249–262. 10.1007/s10658-009-9537-9

[B54] Leite L. N. De Haan E. Krijger M. Kastelein P. van Der Zouwen P. van Den Bovenkamp G. (2014). First report of potato blackleg caused by *Pectobacterium carotovorum* subsp. *brasiliensis* in the Netherlands. New Dis. Rep. 29:24 10.5197/j.2044-0588.2014.029.024

[B55] Li W. Rokni-Zadeh H. De Vleeschouwer M. Ghequire M. G. K. Sinnaeve D. Xie G.-L. (2013). The antimicrobial compound xantholysin defines a new group of *Pseudomonas* cyclic lipopeptides. PLoS ONE 8:e62946 10.1371/journal.pone.0062946 23690965 PMC3656897

[B56] Loman N. J. Constantinidou C. Chan J. Z. M. Halachev M. Sergeant M. Penn C. W. (2012). High-throughput bacterial genome sequencing: an embarrassment of choice, a world of opportunity. Nat. Rev. Microbiol. 10, 599–606. 10.1038/nrmicro2850 22864262

[B57] Loper J. E. Hassan K. A. Mavrodi D. V. Davis E. W. Lim C. K. Shaffer B. T. (2012). Comparative genomics of plant-associated *Pseudomonas* spp.: insights into diversity and inheritance of traits involved in multitrophic interactions. PLoS Genet. 8:e1002784 10.1371/journal.pgen.1002784 22792073 PMC3390384

[B58] Loper J. E. Henkels M. D. Shaffer B. T. Valeriote F. A. Gross H. (2008). Isolation and identification of rhizoxin analogs from *Pseudomonas fluorescens* Pf-5 by using a genomic mining strategy. Appl. Environ. Microbiol. 74, 3085–3093. 10.1128/AEM.02848-07 18344330 PMC2394923

[B59] Ma B. Hibbing M. E. Kim H. S. Reedy R. M. Yedidia I. Breuer J. (2007). Host range and molecular phylogenies of the soft rot enterobacterial genera *Pectobacterium* and *Dickeya*. Phytopathology 97, 1150–1163. 10.1094/PHYTO-97-9-1150 18944180

[B60] Marchler-Bauer A. Derbyshire M. K. Gonzales N. R. Lu S. Chitsaz F. Geer L. Y. (2014). Cdd: NCBI's conserved domain database. Nucleic Acids Res. 43, D222–D226. 10.1093/nar/gku1221 25414356 PMC4383992

[B61] Martín M. G. Sender P. D. Peirú S. De Mendoza D. Magni C. (2004). Acid-inducible transcription of the operon encoding the citrate lyase complex of *Lactococcus lactis* biovar *diacetylactis* CRL264. J. Bacteriol. 186, 5649–5660. 10.1128/JB.186.17.5649-5660.2004 15317769 PMC516808

[B63] Massé E. vanderpool C. K. Gottesman S. (2005). Effect of RyhB small RNA on global iron use in *Escherichia coli*. J. Bacteriol. 187, 6962–6971. 10.1128/JB.187.20.6962-6971.2005 16199566 PMC1251601

[B64] Medema M. H. Blin K. Cimermancic P. De Jager V. Zakrzewski P. Fischbach M. A.. (2011). AntiSMASH: rapid identification, annotation and analysis of secondary metabolite biosynthesis gene clusters in bacterial and fungal genome sequences. Nucleic Acids Res. 39, W339–W346. 10.1093/nar/gkr466 21672958 PMC3125804

[B65] Mercado-Blanco J. (2015). *Pseudomonas* strains that exert biocontrol of plant pathogens, in Pseudomonas: Volume 7: New Aspects of Pseudomonas Biology, ed Ramos J.-L. (Dordrecht: Springer Netherlands), 121–172. 10.1007/978-94-017-9555-5_6

[B66] Meyer J.-M. Stintzi A. (1998). Iron metabolism and siderophores in *Pseudomonas* and related species, in Pseudomonas, ed Montie T. C. (New York, NY: Springer US), 201–243. 10.1007/978-1-4899-0120-0_7

[B67] Meyer M. Dimroth P. Bott M. (1997). *In vitro* binding of the response regulator citb and of its carboxy-terminal domain to A + T-rich DNA target sequences in the control region of the divergent citc and cits operons of *Klebsiella pneumoniae*. J. Mol. Biol. 269, 719–731. 10.1006/jmbi.1997.1076 9223636

[B68] Molina-Santiago C. Udaondo Z. Daddaoua A. Roca A. Martín J. Pérez-Victoria I.. (2015). Efflux pump-deficient mutants as a platform to search for microbes that produce antibiotics. Microb. Biotechnol. 8, 716–725. 10.1111/1751-7915.12295 26059350 PMC4476826

[B69] Mulet M. Gomila M. Lemaitre B. Lalucat J. García-Valdés E. (2012). Taxonomic characterisation of *Pseudomonas* strain L48 and formal proposal of *Pseudomonas entomophila* sp. nov. Syst. Appl. Microbiol. 35, 145–149. 10.1016/j.syapm.2011.12.003 22326814

[B70] Nabhan S. De Boer S. H. Maiss E. Wydra K. (2012). Taxonomic relatedness between *Pectobacterium carotovorum* subsp. *carotovorum, Pectobacterium carotovorum* subsp. *odoriferum* and *Pectobacterium carotovorum* subsp. *brasiliense* subsp. nov. J. Appl. Microbiol. 113, 904–913. 10.1111/j.1365-2672.2012.05383.x 22747943

[B62] Newman D. J. Cragg G. M. (2012). Natural products as sources of new drugs over the 30 years from 1981 to 2010. J. Nat. Prod. 75, 311–335. 10.1021/np200906s 22316239 PMC3721181

[B71] Novik G. Savich V. Kiseleva E. (2015). An insight into beneficial *Pseudomonas* bacteria, in *Microbiology in Agriculture and Human Health*, ed Shah M. M. (InTech), 73–105.

[B72] Ouyang Z. Isaacson R. (2006). Identification and characterization of a novel ABC iron transport system, Fit, in *Escherichia coli*. Infect. Immun. 74, 6949–6956. 10.1128/IAI.00866-06 16982838 PMC1698097

[B73] Palleroni N. J. (2005). Geuns I. *Pseudomonas migula* 1894, 237AL (Nom. Cos,. Opin. 5 of the jud. Comm. 1952, 121), in Bergey's Manual of Systematic Bacteriology, vol. 2B, 2nd Edn., eds Brenner D. J. Castenholz R. W. Garrity G. M. Krieg N. R. Staley J. T. (New York, NY: Springer), 323–379.

[B74] Paulsen I. T. Press C. M. Ravel J. Kobayashi D. Y. Myers G. S. A. Mavrodi D. V.. (2005). Complete genome sequence of the plant commensal *Pseudomonas fluorescens* Pf-5. Nat. Biotechnol. 23, 873–878. 10.1038/nbt1110 15980861 PMC7416659

[B75] Pierson L. S. Pierson E. A. (2010). Metabolism and function of phenazines in bacteria: impacts on the behavior of bacteria in the environment and biotechnological processes. Appl. Microbiol. Biotechnol. 86, 1659–1670. 10.1007/s00253-010-2509-3 20352425 PMC2858273

[B76] Pliego C. Kamilova F. Lugtenberg B. (2011). Plant growth-promoting bacteria: fundamentals and exploitation, in Bacteria in Agrobiology: Crop Ecosystems, ed Maheshwari D. K. (Berlin; Heidelberg: Springer-Verlag), 61–96. 10.1007/978-3-642-18357-7_11

[B77] Potvin E. Sanschagrin F. Levesque R. C. (2008). Sigma factors in *Pseudomonas aeruginosa*. FEMS Microbiol. Rev. 32, 38–55. 10.1111/j.1574-6976.2007.00092.x 18070067

[B78] Raaijmakers J. M. De Bruijn I. de Kock M. J. D. (2006). Cyclic lipopeptide production by plant-associated *Pseudomonas* spp.: diversity, activity, biosynthesis, and regulation. Mol. Plant Microbe Interact. 19, 699–710. 10.1094/MPMI-19-0699 16838783

[B79] Raaijmakers J. M. de Bruijn I. Nybroe O. Ongena M. (2010). Natural functions of lipopeptides from *Bacillus* and *Pseudomonas*: more than surfactants and antibiotics. FEMS Microbiol. Rev. 34, 1037–1062. 10.1111/j.1574-6976.2010.00221.x 20412310

[B80] Raaijmakers J. M. Mazzola M. (2012). Diversity and natural functions of antibiotics produced by beneficial and plant pathogenic bacteria. Annu. Rev. Phytopathol. 50, 403–424. 10.1146/annurev-phyto-081211-172908 22681451

[B81] Raaijmakers J. M. Weller D. M. (1998). Natural plant protection by 2,4-diacetylphloroglucinol-producing *Pseudomonas* spp. In take-all decline soils. Mol. Plant Microbe Interact. 11, 144–152. 10.1094/MPMI.1998.11.2.144

[B83] Raes J. Korbel J. O. Lercher M. J. von Mering C. Bork P. (2007). Prediction of effective genome size in metagenomic samples. Genome Biol. 8:R10. 10.1186/gb-2007-8-1-r10 17224063 PMC1839125

[B84] Ramette A. Frapolli M. Fischer-Le Saux M. Gruffaz C. Meyer J.-M. Défago G.. (2011). *Pseudomonas protegens* sp. nov., widespread plant-protecting bacteria producing the biocontrol compounds 2,4-diacetylphloroglucinol and pyoluteorin. Syst. Appl. Microbiol. 34, 180–188. 10.1016/j.syapm.2010.10.005 21392918

[B85] Raoul des Essarts Y. Cigna J. Quêtu-Laurent A. Caron A. Munier E. Beury-Cirou A.. (2016). Biocontrol of the potato blackleg and soft-rot diseases caused by *Dickeya dianthicola*. Appl. Environ. Microbiol. 82, 268–278. 10.1128/AEM.02525-15 26497457 PMC4702623

[B82] Reese M. G. (2001). Application of a time-delay neural network to promoter annotation in the *Drosophila melanogaster* genome. Comput. Chem. 26, 51–56. 10.1016/S0097-8485(01)00099-7 11765852

[B86] Richter M. Rosselló-Móra R. (2009). Shifting the genomic gold standard for the prokaryotic species definition. Proc. Natl. Acad. Sci. U.S.A. 106, 19126–19131. 10.1073/pnas.0906412106 19855009 PMC2776425

[B87] Rokni-Zadeh H. Mangas-Losada A. de Mot R. (2011). PCR detection of novel non-ribosomal peptide synthetase genes in lipopeptide-producing *Pseudomonas*. Microb. Ecol. 62, 941–947. 10.1007/s00248-011-9885-9 21647696

[B88] Sadikot R. T. Blackwell T. S. Christman J. W. Prince A. S. (2005). Pathogen-host interactions in *Pseudomonas aeruginosa* pneumonia. Am. J. Respir. Crit. Care Med. 171, 1209–1223. 10.1164/rccm.200408-1044SO 15695491 PMC2718459

[B89] Schulz-Bohm K. Zweers H. de Boer W. Garbeva P. (2015). A fragrant neighborhood: volatile mediated bacterial interactions in soil. Front. Microbiol. 6:1212. 10.3389/fmicb.2015.01212 26579111 PMC4631045

[B90] Schwyn B. Neilands J. B. (1987). Universal chemical assay for the detection and determination of siderophores. Anal. Biochem. 160, 46–56. 10.1016/0003-2697(87)90612-9 2952030

[B91] Sievers F. Wilm A. Dineen D. Gibson T. J. Karplus K. Li W.. (2011). Fast, scalable generation of high-quality protein multiple sequence alignments using clustal omega. Mol. Syst. Biol. 7, 539–539. 10.1038/msb.2011.75 21988835 PMC3261699

[B92] Silby M. W. Winstanley C. Godfrey S. A. Levy S. B. Jackson R. W. (2011). *Pseudomonas* genomes: diverse and adaptable. FEMS Microbiol. Rev. 35, 652–680. 10.1111/j.1574-6976.2011.00269.x 21361996

[B93] Sławiak M. Łojkowska E. van der Wolf J. M. (2009). First report of bacterial soft rot on potato caused by *Dickeya* sp. (syn. *Erwinia chrysanthemi*) in Poland. Plant Pathol. 58, 794–794. 10.1111/j.1365-3059.2009.02028.x

[B94] Solovyev V. Salamov A. (2011). Automatic annotation of microbial genomes and metagenomic sequences, in Metagenomics and its Applications in Agriculture, Biomedicine and Environmental Studies, ed Li R. W. (Nova Science Publishers), 61–78.

[B95] Song C. Schmidt R. De Jager V. Krzyzanowska D. Jongedijk E. Cankar K.. (2015). Exploring the genomic traits of fungus-feeding bacterial genus *Collimonas*. BMC Genomics 16:1103. 10.1186/s12864-015-2289-3 26704531 PMC4690342

[B96] Stutz E. W. (1986). Naturally occurring fluorescent pseudomonads involved in suppression of black root rot of tobacco. Phytopathology 76, 181–181. 10.1094/Phyto-76-181

[B97] Takeuchi K. Noda N. Someya N. (2014). Complete genome sequence of the biocontrol strain *Pseudomonas protegens* Cab57 discovered in japan reveals strain-specific diversity of this species. PLoS ONE 9, e93683. 10.1371/journal.pone.0093683 24695768 PMC3973561

[B98] Tamura K. Stecher G. Peterson D. Filipski A. Kumar S. (2013). Mega6: molecular evolutionary genetics analysis version 6.0. Mol. Biol. Evol. 30, 2725–2729. 10.1093/molbev/mst197 24132122 PMC3840312

[B99] Thoma S. Schobert M. (2009). An improved *Escherichia coli* donor strain for diparental mating. FEMS Microbiol. Lett. 294, 127–132. 10.1111/j.1574-6968.2009.01556.x 19431232

[B100] Toth I. K. van der Wolf J. M. Saddler G. Lojkowska E. Hélias V. Pirhonen M. (2011). *Dickeya* species: an emerging problem for potato production in Europe. Plant Pathol. 60, 385–399. 10.1111/j.1365-3059.2011.02427.x

[B101] Tsror L. Erlich O. Lebiush S. Hazanovsky M. Zig U. Sławiak M. (2009). Assessment of recent outbreaks of *Dickeya* sp. (syn. *Erwinia chrysanthemi*) slow wilt in potato crops in Israel. Eur. J. Plant Pathol. 123, 311–320. 10.1007/s10658-008-9368-0

[B102] Tvrzová L. Schumann P. Spröer C. Sedlácek I. Pácová Z. Sedo O.. (2006). *Pseudomonas moraviensis* sp. nov. and *Pseudomonas vranovensis* sp. nov., soil bacteria isolated on nitroaromatic compounds, and emended description of *Pseudomonas asplenii*. Int. J. Syst. Evol. Microbiol. 56, 2657–2663. 10.1099/ijs.0.63988-0 17082407

[B103] Uchino M. Shida O. Uchimura T. Komagata K. (2001). Recharacterization of *Pseudomonas fulva* Iizuka and Komagata 1963, and proposals of *Pseudomonas parafulva* sp. nov. and *Pseudomonas cremoricolorata* sp. nov. J. Gen. Appl. Microbiol. 47, 247–261. 10.2323/jgam.47.247 12483612

[B104] van der Merwe J. J. Coutinho T. A. Korsten L. van der Waals J. E. (2009). *Pectobacterium carotovorum* subsp. *brasiliensis* causing blackleg on potatoes in South Africa. Eur. J. Plant. Pathol. 126, 175–185. 10.1007/s10658-009-9531-2

[B105] van der Wolf J. M. Nijhuis E. H. Kowalewska M. J. Saddler G. S. Parkinson N. Elphinstone J. G.. (2014). *Dickeya solani* sp. nov., a pectinolytic plant-pathogenic bacterium isolated from potato (*Solanum tuberosum*). Int. J. Syst. Evol. Microbiol. 64, 768–774. 10.1099/ijs.0.052944-0 24225027

[B106] Visca P. Imperi F. Lamont I. L. (2007). *Pyoverdine siderophores*: from biogenesis to biosignificance. Trends Microbiol. 15, 22–30. 10.1016/j.tim.2006.11.004 17118662

[B107] Vodovar N. Vinals M. Liehl P. Basset A. Degrouard J. Spellman P.. (2005). Drosophila host defense after oral infection by an entomopathogenic *Pseudomonas* species. Proc. Natl. Acad. Sci. U.S.A. 102, 11414–11419. 10.1073/pnas.0502240102 16061818 PMC1183552

[B108] Weber T. Marahiel M. A. (2001). Exploring the domain structure of modular nonribosomal peptide synthetases. Structure 9, R3–R9. 10.1016/S0969-2126(00)00560-8 11342140

[B109] Weller D. M. (2007). *Pseudomonas* biocontrol agents of soilborne pathogens: looking back over 30 years. Phytopathology 97, 250–256. 10.1094/PHYTO-97-2-0250 18944383

[B110] Wu X. Monchy S. Taghavi S. Zhu W. Ramos J. van der Lelie D. (2011). Comparative genomics and functional analysis of niche-specific adaptation in *Pseudomonas putida*. FEMS Microbiol. Rev. 35, 299–323. 10.1111/j.1574-6976.2010.00249.x 20796030 PMC3056050

[B111] Ye L. Hildebrand F. Dingemans J. Ballet S. Laus G. Matthijs S.. (2014). Draft genome sequence analysis of a *Pseudomonas putida* W15Oct28 strain with antagonistic activity to Gram-positive and *Pseudomonas* sp. pathogens. PLoS ONE 9:e110038. 10.1371/journal.pone.0110038 25369289 PMC4219678

[B112] Young J. M. (2010). Taxonomy of *Pseudomonas syringae*. J. Plant. Pathol. 92, S95–S14. 10.4454/jpp.v92i1sup.2501

[B113] Yu X. Chen M. Jiang Z. Hu Y. Xie Z. (2014). The two-component regulators GacS and GacA positively regulate a nonfluorescent siderophore through the gac/rsm signaling cascade in high-siderophore-yielding *Pseudomonas* sp. strain HYS. J. Bacteriol. 196, 3259–3270. 10.1128/jb.01756-14 24982309 PMC4135692

[B114] Zhou Y. Liang Y. Lynch K. H. Dennis J. J. Wishart D. S. (2011). Phast: a fast phage search tool. Nucleic Acids Res. 39, W347–W352. 10.1093/nar/gkr485 21672955 PMC3125810

