# Supplementary material for: When Genome-Based Approach Meets the “Old but Good”: Revealing Genes Involved in the Antibacterial Activity of Pseudomonas sp. P482 against Soft Rot Pathogens
Source: Front Microbiol. 2016 May 26;7:782. doi: 10.3389/fmicb.2016.00782 (PMC4880745; doi:10.3389/fmicb.2016.00782)
Supplement: Supplementary file 1 [file Table1.DOCX]

Supplementary Material

**When genome-based approach meets the ‘old but good’: revealing genes involved in the antibacterial activity of *Pseudomonas* sp. P482 against soft rot pathogens**

Dorota M. Krzyżanowska^1^, Adam Ossowicki^1^, Magdalena Rajewska^1^, Tomasz Maciąg^1^, Magdalena Jabłońska^1^, Michał Obuchowski^2^, Stephan Heeb^3^, and Sylwia Jafra^1,*^

*** Correspondence:** Sylwia Jafra, [sylwia.jafra@biotech.ug.edu.pl](mailto:sylwia.jafra@biotech.ug.edu.pl)

**Supplementary Tables**

# Table S1. Accession numbers of genes used for phylogenetic study based on16S rRNA gene analysis and MLSA.

| ***Strain*** | **Gene/Sequence** | **Accession numer ^A^** |
| --- | --- | --- |
| ***16S rRNA*** |  |  |
| *Pseudomonas* sp. P482 | 16S rRNA (*rrs*) | JHTS00000000 |
| *P. donghuensis* HYS^T^ | 16S rRNA (*rrs*) | NZ_JH650764.1 |
| *P. aeruginosa* SNP 0614^T^ | 16S rRNA (*rrs*) | NR_118644.1 |
| *P. vranovensis* DSM 16006^T^ = 2B2^T^ | 16S rRNA (*rrs*) | NR_043313.1 |
| *P. asplenii* CCM 7744^T^ = ATCC 23835^T^ | 16S rRNA (*rrs*) | NR_040802.1 |
| *P. oryzihabitans* L-1^T^ | 16S rRNA (*rrs*) | NR_025881.1 |
| *P. entomophila* L48^T^ | 16S rRNA (*rrs*) | NR_102854.1 |
| *P. putida* DSM 291^T^ = ATCC12633^T^ | 16S rRNA (*rrs*) | NR_114479.1 |
| *P. japonica* NBRC 103040^T^ | 16S rRNA (*rrs*) | NR_040992.1 |
| *P. mosselii* CFML 90-83^T^ | 16S rRNA (*rrs*) | NR_024924.1 |
| *P. plecoglossicida* FPC 951^T^ | 16S rRNA (*rrs*) | NR_024662.1 |
| *P. graminis* DSM 11363^T^ | 16S rRNA (*rrs*) | NR_026395.1 |
| *P. lutea* OK2^T^ | 16S rRNA (*rrs*) | NR_029103.1 |
| *P. monteilii* NBRC 103158^T^ = CIP104883^T^ | 16S rRNA (*rrs*) | NR_112073.1 |
| *P. cremoricolorata* DSM 17059^T^ = IAM 1541^T^ | 16S rRNA (*rrs*) | NR_040860.1 |
| *P. moraviensis* 1B4^T^ | 16S rRNA (*rrs*) | NR_043314.1 |
| *P. rhizosphaerae* IH5^T^ | 16S rRNA (*rrs*) | NR_029063.1 |
| *P. parafulva* AJ2129^T^ | 16S rRNA (*rrs*) | NR_104278.1 |
| *P. chengduensis* MBR^T^ | 16S rRNA (*rrs*) | NR_125523.1 |
| *P. taiwanensis* BCRC 17751^T^ | 16S rRNA (*rrs*) | NR_116172.1 |
| *P. stutzeri* ATCC 17588^T^ | 16S rRNA (*rrs*) | NR_103934.1 |
| *P. punonensis* LMT03^T^ | 16S rRNA (*rrs*) | NR_109583.1 |
| *P. veronii* CIP 104663^T^ | 16S rRNA (*rrs*) | NR_028706.1 |
| *P. baetica* a390^T^ | 16S rRNA (*rrs*) | NR_116899.1 |
| *P. fluorescens* CCM 2115^T^ | 16S rRNA (*rrs*) | NR_115715.1 |
| *P. vancouverensis* DhA-51^T^ | 16S rRNA (*rrs*) | NR_041953.1 |
| *P. benzenivorans* DSM 8628^T^ | 16S rRNA (*rrs*) | NR_116904.1 |
| *P. antarctica* CMS 35^T^ | 16S rRNA (*rrs*) | NR_025586.1 |
| *P. migulae* CIP 105470^T^ | 16S rRNA (*rrs*) | NR_024927.1 |
| *P. rhodesiae* CIP 104664^T^ | 16S rRNA (*rrs*) | NR_024911.1 |
| *P. meridiana* CMS 38^T^ | 16S rRNA (*rrs*) | NR_025587.1 |
| *P. orientalis* CFML 96-170^T^ | 16S rRNA (*rrs*) | NR_024909.1 |
| *P. alcaliphila* AL15-21^T^ | 16S rRNA (*rrs*) | NR_024734.1 |
| *P. trivialis* P513/19^T^ | 16S rRNA (*rrs*) | NR_028987.1 |
| *P. tolaasii* LMG 2342^T^ | 16S rRNA (*rrs*) | NR_041799.1 |
| *P. poae* P527/13^T^ | 16S rRNA (*rrs*) | NR_028986.1 |
| *P. marginalis* ATCC 10844^T^ | 16S rRNA (*rrs*) | NR_112072.1 |
| *P. kuykendallii* H2^T^ | 16S rRNA (*rrs*) | NR_118155.1 |
| *P. chlororaphis* ATCC 9446^T^ | 16S rRNA (*rrs*) | NR_116763.1 |
| *P. cedrina* CFML 96-198^T^ | 16S rRNA (*rrs*) | NR_024912.1 |
| *P. asturiensis* LPPA221^T^ | 16S rRNA (*rrs*) | NR_108461.1 |
| *P. kilonensis* 520-20^T^ | 16S rRNA (*rrs*) | NR_028929.1 |
| *P. luteola* 4239^T^ | 16S rRNA (*rrs*) | NR_037134.1 |
| *P. lurida* P513/18^T^ | 16S rRNA (*rrs*) | NR_042199.1 |
| *P. costantinii* CFBP 5705^T^ | 16S rRNA (*rrs*) | NR_025164.1 |
| *P. libanensis* CIP 105460^T^ | 16S rRNA (*rrs*) | NR_024901.1 |
| *P. proteolytica* CMS 64^T^ | 16S rRNA (*rrs*) | NR_025588.1 |
| *P. xanthomarina* KMM 1447^T^ | 16S rRNA (*rrs*) | NR_041044.1 |
| *P. koreensis* Ps9-14^T^ | 16S rRNA (*rrs*) | NR_025228.1 |
| *P. synxantha* IAM 12356^T^ | 16S rRNA (*rrs*) | NR_043425.1 |
| *P. fuscovaginae* ICMP 5940^T^ | 16S rRNA (*rrs*) | NR_116700.1 |
| *P. protegens* CHAO^T^ | 16S rRNA (*rrs*) | NR_114749.1 |
| ***MLSA*** |  |  |
| *Pseudomonas sp. P482* | *gyrB* | JHTS00000000 |
| *Pseudomonas sp. P482* | *rpoB* | JHTS00000000 |
| *Pseudomonas sp. P482* | *rpoD* | JHTS00000000 |
| *P. vranovensis* DSM 16006^T^ = 2B2^T^ | *gyrB* | HE577791.1 |
| *P. vranovensis* DSM 16006^T^ = 2B2^T^ | *rpoB* | HE577799.1 |
| *P. vranovensis* DSM 16006^T^ = 2B2^T^ | *rpoD* | HE577793.1 |
| *P. donghuensis* HYS^T^ | *gyrB* | AJJP01000000 |
| *P. donghuensis* HYS^T^ | *rpoB* | AJJP01000000 |
| *P. donghuensis* HYS^T^ | *rpoD* | AJJP01000000 |
| *Cellvibrio japonicus* Ueda107^T^ | *gyrB* | CP000934.1 |
| *C. japonicus* Ueda107^T^ | *rpoB* | CP000934.1 |
| *C. japonicus* Ueda107^T^ | *rpoD* | CP000934.1 |
| *P. fuscovaginae* ICMP5940^T^ | *gyrB* | NZ_BATG00000000.1 |
| *P. fuscovaginae* ICMP5940^T^ | *rpoB* | NZ_BATG00000000.1 |
| *P. fuscovaginae* ICMP5940^T^ | *rpoD* | NZ_BATG00000000.1 |
| *P. asplenii* CCM 7744^T^ = ATCC 23835^T^ | *gyrB* | Unpublished data |
| *P. asplenii* CCM 7744^T^ = ATCC 23835^T^ | *rpoB* | Unpublished data |
| *P. asplenii* CCM 7744^T^ = ATCC 23835^T^ | *rpoD* | Unpublished data |
| *P. putida* DSM 291^T^ = ATCC12633^T^ | *gyrB* | AP013070.1 |
| *P. putida* DSM 291^T^ = ATCC12633^T^ | *rpoB* | AP013070.1 |
| *P. putida* DSM 291^T^ = ATCC12633^T^ | *rpoD* | AP013070.1 |

^A^ For the sequences retrieved from draft genomes, the accession numbers of the whole-genome shotgun sequencing projects are provided (master records).
