# Supplementary material for: When Genome-Based Approach Meets the “Old but Good”: Revealing Genes Involved in the Antibacterial Activity of Pseudomonas sp. P482 against Soft Rot Pathogens
Source: Front Microbiol. 2016 May 26;7:782. doi: 10.3389/fmicb.2016.00782 (PMC4880745; doi:10.3389/fmicb.2016.00782)
Supplement: Supplementary file 2 [file Table2.DOCX]

Supplementary Material

**When genome-based approach meets the ‘old but good’: revealing genes involved in the antibacterial activity of *Pseudomonas* sp. P482 against soft rot pathogens**

Dorota M. Krzyżanowska^1^, Adam Ossowicki^1^, Magdalena Rajewska^1^, Tomasz Maciąg^1^, Magdalena Jabłońska^1^, Michał Obuchowski^2^, Stephan Heeb^3^, and Sylwia Jafra^1,*^

*** Correspondence:** Sylwia Jafra, [sylwia.jafra@biotech.ug.edu.pl](mailto:sylwia.jafra@biotech.ug.edu.pl)

**Supplementary Tables**

# Table S2. The GenBank/EMBL/DDBJ accession numbers of genomic sequences used in this study.

| **Strain** | **Accesion numer** | **Genome status**  **(contig number)^A^** | **Genome size [Mbp]** |
| --- | --- | --- | --- |
| *Pseudomonas* sp. P482 | JHTS00000000 | D (4/69)^B^ | 5.65 |
| *P. donghuensis* HYS^T^ | AJJP00000000 | D (231) | 5.64 |
| *P. vranovensis* DSM 16006^T^ | AUED00000000 | D (42) | 5.70 |
| *P. entomophila* L48^T^ | CT573326 | C | 5.89 |
| *P. putida* DSM 291^T^ | AP013070 | C | 6.16 |
| *P. monteilii* NBRC 103158^T^ | ASM62124v1 | C | 6.31 |
| *P. putida* KT2440 | AE015451 | C | 6.18 |
| *P. cremoricolorata* DSM 17059^T^ | AUEA00000000 | D (27) | 4.09 |
| *P. protegens* CHA0^T^ | NC_021237 | C | 6.87 |
| *P. asplenii* CCM 7744^T^ | unpublished ^C^ | D (69) | 6.40 |
| *P. fuscovaginae* ICMP 5940^T^ | NZ_BATG01000000 | D (459) | 6.38 |
| *P. moraviensis* DSM 16007^T^ | AYMZ00000000 | D (13) | 6.30 |

^A^ D – draft genome; C – complete chromosome

^B^ the genome of *P. asplenii* CCM 7744^T^ is to be published elsewhere
