# Supplementary material for: When Genome-Based Approach Meets the “Old but Good”: Revealing Genes Involved in the Antibacterial Activity of Pseudomonas sp. P482 against Soft Rot Pathogens
Source: Front Microbiol. 2016 May 26;7:782. doi: 10.3389/fmicb.2016.00782 (PMC4880745; doi:10.3389/fmicb.2016.00782)
Supplement: Supplementary file 3 [file Table3.DOCX]

Supplementary Material

**When genome-based approach meets the ‘old but good’: revealing genes involved in the antibacterial activity of *Pseudomonas* sp. P482 against soft rot pathogens**

Dorota M. Krzyżanowska^1^, Adam Ossowicki^1^, Magdalena Rajewska^1^, Tomasz Maciąg^1^, Magdalena Jabłońska^1^, Michał Obuchowski^2^, Stephan Heeb^3^, and Sylwia Jafra^1,*^

*** Correspondence:** Sylwia Jafra, [sylwia.jafra@biotech.ug.edu.pl](mailto:sylwia.jafra@biotech.ug.edu.pl)

**Supplementary Tables**

# Table S3. List of biologically active metabolites produced by *Pseudomonas* spp. and the results of manual search of the P482 genome towards the presence of genes involved in the production of these compounds.

| **Metabolite and fearures** | **Protein sequence used as query: name, length and the strain of origin** | **ORF encoding the best hit. Identity/similarity values [%] and the query coverage (qq)^A^** |
| --- | --- | --- |
| ***Antibiotics* ^B^** |  |  |
| **2,4-Diacetylphloroglucinol**  polyketide | PhlD (AAY95147), 349 aa  *P. protegens* Pf-5 | **No^C^**  BV82_4402  36%/54%, qq 13% |
| **2,5-Dialkylresorcinols**  polyketide | DarA (AAN18031), 313 aa  *P. chlororaphis* subsp. *aurantiaca* | **No**  BV82_5133  28%/ 45%, qq 26% |
| **2-hexyl, 5-propyl resorcinol** polyketide | “dialkyl resorcinol condensing enzyme” (AFK32664), 313 aa  *P. fluorescens* PCL 1606 | **No**  BV82_1607  43%/ 68%, qq 10% |
| **Quinolones** | PqsD (P20582), 337 aa  *P. aeruginosa* PAO1 | **No**  BV82_867  28%/ 40%, qq 95% |
| **Hydrogen cyanide (HCN)**  volatile toxic compound | HcnB (YP_610913), 464 aa  *P. entomophila* L48 | **Yes**  BV82_2273  82%/ 88%, qq 100%  phenotype also positive^D^ |
| **Phenazines**  alcaloids | PhzD (ADP21172), 204 aa  *P. chlororaphis* subsp*. chlororaphis* GP72 | **No**  BV82_4148  25%/ 45%, qq 71% |
| **Xantholysin**  cyclic lipopeptide | XtlA (AGM14925), 2112 aa  *P. putida* BW11M1 | **No**  P482_1009  31%/ 43%, qq 104% ^A^ |
| **Massetolides**  cyclic lipopeptides | MassB (ABH06368), 4307 aa  *P. fluorescens* SS101 | **No**  BV82_1009  31%/ 45%, qq 58% |
| **Mupirocin**  polyketide | MmpD (AAM12913.2), 6522 aa  *P. fluorescens* NCIMB 10586 | **No**  BV82_4054  30%/ 43%, qq 4% |
| **Orfamides**  cyclic lipopeptides | OfaC (AAY91421.3), 4901 aa  *P. protegens* Pf-5 | **No**  BV82_1009  37%/ 52%, qq 49% |
| **Pyoluteorin**  polyketide | PltB (AAC38075), 2458 aa  *P. protegens* Pf-5 | **No**  BV82_4054  28%/ 45%, qq 15% |
| **Pyrrolnitrin**  aminoacid derivative | PrnB (AAY92871), 361 aa  *P. protegens* Pf-5 | **No**  BV82_1009  39%/ 56%, qq 11% |
| **Rhizoxins**  nonribosomal peptides-polyketides | RzxE (AAY92265), 4163 aa  *P. protegens* Pf-5 | **No**  BV82_4054  24%/ 42%, qq 9% |
| **Syringopeptin**  cyclic lipopeptide | Syringopeptin synthetase B (ELS42846), 5486 aa  *P. syringae* pv. *syringae* B64 | **No**  BV82_1009  31%/ 46%, qq 54% |
| **Syringomycin**  cyclic lipopeptide | Syringomycin biosynthesis enzyme 1 (EXL31113), 614 aa  *P. syringae* pv. *syringae* str. B301D-R | **No**  BV82_1009  34%/ 52%, qq 99,7% |
| **Viscosin**  cyclic lipopeptide | ViscB (ESW57243), 4307 aa  *P. fluorescens* BBc6R8 | **No**  BV82_1009  30%/ 43%, qq 47% |
| ***Siderophores*** |  |  |
| **Achromobactin**  citrate-based siderophore | AcsD (EJL06234), 600 aa  *P. chlororaphis* subsp. *aureofaciens* 30-84 | **No**  BV82_3452  27%/ 60%, qq 7% |
| **Quinolobactin/ tioquinolobactin**  derivative of xanthurenic acid | QbsL (AAL65279), 905 aa  *P. fluorescens* ATCC 17400 | **No**  BV82_2127  28%/ 42%, qq 52% |
| **Nonfluorescent siderophore**  not determined | Protein product of Scaffold1 (JH650757.1), position 94935- 96077,  380 aa  *P. donghuensis* HYS^T^ | **Yes**  BV82_4709  100%/ 100%, qq 100% |
| **Pyoverdin**  nonribosomal peptide | PvdD (AAY93354), 4410 aa  *P. protegens* Pf-5 | **Yes**  BV82_3757  51% /65%, qq 58%  phenotype also positive ^E^ |
| **Pseudomonin**  nonribosomal peptide | PmsB (CAA70531), 111 aa  *P. fluorescens* WCS374 | **No**  BV82_3498  30%/ 50%, qq 86% |
| **Pyochelin**  nonribosomal peptide | PchD (CAA57966), 546 aa  *P. aeruginosa* PAO1 | **No**  BV82_721  25%/ 43%, qq 66% |
| ***Biosurfactants*** |  |  |
| **Arthrofactin**  nonribosomal peptide | ArfC (BAC67536), 5924 aa  *P.* sp. MIS38 | **No**  BV82_1009  36%/ 51%, qq 42% |
| **Putisolvin**  nonribosomal peptide | PsoB (ABW17376), 7474 aa  *P. putida* PCL1445 | **No**  BV82_1009  32%/ 46%, qq 34% |
| ***Other*** |  |  |
| **Paerucumarin and pseudoverdin** | PvcD (AAC21674), 215 aa  *P. aeruginosa* PAO1 | **No**  BV82_2369  39%/ 57%, qq 81% |

^A^ query coverage (qq) – here the length of the subject sequence aligned to the query sequence, divided by the length of the query

^B^ antibiotics – antibacterial and/or antifungal compounds

^C^ the cutoff value for positive hits were query coverage and identity values both ≥50%

^D^ HCN production determined by a colorimetric assay (Castric & Castric 1983) (A. Ossowicki, unpublished data)

^E^ strong yellow fluorescence on King’s B medium

Castric, K.F. & Castric, P.A., 1983. Method for Rapid Detection of Cyanogenic Bacteria. *Appl. Envir. Microbiol.*, 45(2), pp.701–702.
