# Supplementary material for: When Genome-Based Approach Meets the “Old but Good”: Revealing Genes Involved in the Antibacterial Activity of Pseudomonas sp. P482 against Soft Rot Pathogens
Source: Front Microbiol. 2016 May 26;7:782. doi: 10.3389/fmicb.2016.00782 (PMC4880745; doi:10.3389/fmicb.2016.00782)
Supplement: Supplementary file 4 [file Table4.pdf]

Supplementary Material

When genome-based approach meets the 'old but good': revealing genes involved in the antibacterial activity of *Pseudomonas* sp. P482 against soft rot pathogens

Dorota M. Krzyżanowska[1], Adam Ossowicki[1], Magdalena Rajewska[1], Tomasz Maciąg[1], Magdalena Jabłońska[1], Michał Obuchowski[2], Stephan Heeb[3], and Sylwia Jafra[1,*]

*** Correspondence:** Sylwia Jafra, sylwia.jafra@biotech.ug.edu.pl

Supplementary Tables

Table S4. PCR primers designed and used in this study.

Name	Sequence	Introduced cloning site
**F_ XbaI_P482_1009**	5'AGCCAGTCTAGAAAGCAACTGGCCGCTTATCT	XbaI
**R_ XhoI_P482_1009**	5'GACGAACTCGAGGACGGTCTGATGCTGGAACA	XhoI
**F_XbaI_482_3755_new**	5'AGCCAGTCTAGATGCTTTCCTATGCCCAGCAA	XbaI
**R_XhoI_482_3755_new**	5'GACGAACTCGAGCGGCATAGGTTTGCATCAGC	XhoI
**F_XbaI_P482_4705**	5'TTAAATCTAGATCGTGGTCAAGCACGGTTAC	XbaI
**R_XhoI_P482_4705**	5'TTAAACTCGAGCGCGCTTTGTACTGATGCTC	XhoI
**F_XbaI_P482_4706_B**	5'ATTATTCTAGAACTAGGCACTTCCACCACCA	XbaI

**R_XhoI_P482_4706_B**	5’TTAAACTCGAGTGCCGGTACCGAACTCTACT	XhoI
**F_XbaI_P482_4709**	5’AGTAAGTCTAGACCGCTGTATTTCGACAACCT	XbaI
**R_XhoI_P482_4709**	5’GACGAACTCGAGGATGTCGCCTTCGTGGTAAC	XhoI
**tpnRL13–2_F_LONG**	5’ATGAGTCAGCAACACCTTCTTCACGA	-
**tpnRL17–1_R_LONG**	5’TGGACAACAAGCCAGGGATGTAACG	-
**F_pKNOCK_backbone**	5’GGTGCCCTGAATGACTCCA	-
**R_pKNOCK_backbone**	5’AAAAGCGGCCATTTTCCACC	-
**F_outof_pKNOCK**	5’CACGTACTAAGCTCTCATGTTTGAACA	-

All oligonucleotides were synthesized by Genomed (Warsaw, Poland). The sequences recognized by the XbaI or the XhoI endonucleases are underlined.
