# Supplementary material for: When Genome-Based Approach Meets the “Old but Good”: Revealing Genes Involved in the Antibacterial Activity of Pseudomonas sp. P482 against Soft Rot Pathogens
Source: Front Microbiol. 2016 May 26;7:782. doi: 10.3389/fmicb.2016.00782 (PMC4880745; doi:10.3389/fmicb.2016.00782)
Supplement: Supplementary file 5 [file Table5.DOCX]

Supplementary Material

**When genome-based approach meets the ‘old but good’: revealing genes involved in the antibacterial activity of *Pseudomonas* sp. P482 against soft rot pathogens**

Dorota M. Krzyżanowska^1^, Adam Ossowicki^1^, Magdalena Rajewska^1^, Tomasz Maciąg^1^, Magdalena Jabłońska^1^, Michał Obuchowski^2^, Stephan Heeb^3^, and Sylwia Jafra^1,*^

*** Correspondence:** Sylwia Jafra, [sylwia.jafra@biotech.ug.edu.pl](mailto:sylwia.jafra@biotech.ug.edu.pl)

**Supplementary Tables**

# Table S5. Primer pairs and annealing temperatures applied for PCR and the expected lengths of the amplified products.

| **Target/Locus** | **Primer pair used for amplification** | **Annealing temperature for PCR** | **Size of the amplified gene fragment (bp)** |
| --- | --- | --- | --- |
| BV82_1009 | F_XbaI_482_1009 / R_XhoI_482_1009 | 60°C | 417 |
| BV82_3755 | F_XbaI_482_3755_new / R_XhoI_482_3755_new | 60°C | 453 |
| BV82_4705 | F_XbaI_P482_4705 / R_XhoI_P482_4705 | 60°C | 322 |
| BV82_4706 | F_XbaI_P482_4706_B / R_XhoI_P482_4706_B | 60°C | 316 |
| BV82_4709 | F_XbaI_P482_4709 / R_XhoI_P482_4709 | 64°C | 417 |
| Backbone of the pKNOCK vector | F_pKNOCK_backbone / R_pKNOCK_backbone | 60°C | 448 |
