# Supplementary material for: When Genome-Based Approach Meets the “Old but Good”: Revealing Genes Involved in the Antibacterial Activity of Pseudomonas sp. P482 against Soft Rot Pathogens
Source: Front Microbiol. 2016 May 26;7:782. doi: 10.3389/fmicb.2016.00782 (PMC4880745; doi:10.3389/fmicb.2016.00782)
Supplement: Supplementary file 6 [file Table6.pdf]

Supplementary Material

When genome-based approach meets the 'old but good': revealing genes involved in the antibacterial activity of *Pseudomonas* sp. P482 against soft rot pathogens

Dorota M. Krzyżanowska[1], Adam Ossowicki[1], Magdalena Rajewska[1], Tomasz Maciąg[1], Magdalena Jabłońska[1], Michał Obuchowski[2], Stephan Heeb[3], and Sylwia Jafra[1,*]

*** Correspondence:** Sylwia Jafra, sylwia.jafra@biotech.ug.edu.pl

Supplementary Tables

Table S6. Comparison of biochemical traits between strains *Pseudomonas* sp. P482 and *P. donghuensis* HYS^T. Results were obtained using the API 20NE and API 50CH tests, both performed in duplicate.

		HYS^T	P482
Enzymatic activity:	reduction of nitrates to nitrites	+	+
	reduction of nitrates to nitrogen	-	-
	indole production	-	-
	fermentation of glucose	-	-
	arginine dihydrolase	+	+
	urease	-	-
	hydrolysis of esculin (β-glucosidase)	-	-
	hydrolysis of gelatin (protease)	+	+
	β-galactosidase (para-nitrophenyl-ß-D-galactopyranosidase)	-	-
	cytochrome oxidase	+	+
Assimilation of:	glycerol	+	+
	erythritol	-	-

d-arabinose	-	-
l-arabinose	+	-
d-ribose	-	-
d-xylose	+	+
l-xylose	-	-
d-adonitol	-	-
methyl-ßd-xylopyranoside	-	-
D-galactose	w	-
D-glucose	+	+
D-fructose	+	+
D-mannose	+	+
L-sorbose	-	-
L-rhamnose	-	-
dulcitol	-	-
inositol	-	-
D-mannitol	-	-
D-sorbitol	-	-
methyl-αd-mannopyranoside	-	-
methyl-αd-glucopyranoside	-	-
amygdalin	-	-
arbutin	-	-
esculin ferric citrate	-	-
salicin	-	-

D-cellobiose	-	-
D-maltose	-	-
D-lactose (bovine origin)	-	-
D-melibiose	-	-
D-saccharose (sucrose)	+	+
D-trehalose	-	-
inulin	-	-
D-melezitose	-	-
D-raffinose	-	-
amidon (starch)	-	-
glycogen	-	-
xylitol	-	-
gentiobiose	-	-
D-turanose	-	-
D-lyxose	-	-
D-tagatose	-	-
D-fucose	+	+
L-fucose	-	-
D-arabitol	-	-
L-arabitol	-	-
potassium gluconate	-	-
potassium 2-ketogluconate	-	-
potassium 5-ketogluconate	-	-
N-acetyl-glucosamine	w	-

potassium gluconate	+	+
capric acid	+	+
adipic acid	-	-
malate	+	+
trisodium citrate	+	+
phenylacetic acid	+	+

‘+’ and ‘-‘ stand for ‘positive’ and ‘negative’ reaction respectively; ‘w’ – stands for ‘weak’ (with respect to the other strain tested).
