# Supplementary material for: When Genome-Based Approach Meets the “Old but Good”: Revealing Genes Involved in the Antibacterial Activity of Pseudomonas sp. P482 against Soft Rot Pathogens
Source: Front Microbiol. 2016 May 26;7:782. doi: 10.3389/fmicb.2016.00782 (PMC4880745; doi:10.3389/fmicb.2016.00782)
Supplement: Supplementary file 7 [file Table7.DOCX]

Supplementary Material

**When genome-based approach meets the ‘old but good’: revealing genes involved in the antibacterial activity of *Pseudomonas* sp. P482 against soft rot pathogens**

Dorota M. Krzyżanowska^1^, Adam Ossowicki^1^, Magdalena Rajewska^1^, Tomasz Maciąg^1^, Magdalena Jabłońska^1^, Michał Obuchowski^2^, Stephan Heeb^3^, and Sylwia Jafra^1,*^

*** Correspondence:** Sylwia Jafra, [sylwia.jafra@biotech.ug.edu.pl](mailto:sylwia.jafra@biotech.ug.edu.pl)

**Supplementary Tables**

# Table S7. Genome regions identified by PHAST as containing genetic elements characteristic for prophages.

| **Completeness** | **Contig** | **GenBank accession** | **Region** | **Region length (kb)** | **G+C (%)^A^** | **Possible phage (GenBank accession for phage genome)** | **Included ORFs** | **ORF count** |
| --- | --- | --- | --- | --- | --- | --- | --- | --- |
| intact | P482.contig.3_2 | JHTS01000039.1 | 54075 to 102713 | 48.6 | 58.41 | *Pseudomonas* phage phiPSA1 (NC_024365) | BV82_3043 to BV82_3094 | 61 |
| intact | P482.contig.1_9 | JHTS01000013.1 | 129974 to 155937 | 25.9 | 61.15 | *Escherichia* phage vB_EcoM-ep3 (NC_025430) | BV82_1280 to BV82_1307 | 29 |
| questionable | P482.contig.1_5 | JHTS01000009.1 | 350635 to 374993 | 24.3 | 60.80 | *Escherichia* phage vB_EcoM-ep3 (NC_025430) | BV82_0560 to BV82_0585 | 25 |
| incomplete | P482.contig.1_5 | JHTS01000009.1 | 4633 to 37293 | 32.6 | 61.88 | *Acanthamoeba polyphaga* mimivirus (NC_014649) | BV82_0241 to BV82_0274 | 21 |

^A^ the average G+C content for the draft genome of P482 is 62.38%
