# Supplementary material for: When Genome-Based Approach Meets the “Old but Good”: Revealing Genes Involved in the Antibacterial Activity of Pseudomonas sp. P482 against Soft Rot Pathogens
Source: Front Microbiol. 2016 May 26;7:782. doi: 10.3389/fmicb.2016.00782 (PMC4880745; doi:10.3389/fmicb.2016.00782)
Supplement: Supplementary file 8 [file Table8.DOCX]

Supplementary Material

**When genome-based approach meets the ‘old but good’: revealing genes involved in the antibacterial activity of *Pseudomonas* sp. P482 against soft rot pathogens**

Dorota M. Krzyżanowska^1^, Adam Ossowicki^1^, Magdalena Rajewska^1^, Tomasz Maciąg^1^, Magdalena Jabłońska^1^, Michał Obuchowski^2^, Stephan Heeb^3^, and Sylwia Jafra^1,*^

*** Correspondence:** Sylwia Jafra, [sylwia.jafra@biotech.ug.edu.pl](mailto:sylwia.jafra@biotech.ug.edu.pl)

**Supplementary Tables**

# Table S8. Genome regions identified by IslandPick as putative genomic islands.

| **No.** | **Contig** | **GenBank accession** | **Range** | **Included ORFs** |
| --- | --- | --- | --- | --- |
| 1 | P482.contig.1_5 | JHTS01000009.1 | 102 to 6149 | BV82_0239 to BV82_0241 |
| 2 | P482.contig.1_9 | JHTS01000013.1 | 213847 to 218895 | BV82_1357 to BV82 1361 |
| 3 | P482.contig.1_12 | JHTS01000016.1 | 34014 to 41041 | BV82_1749 to BV82_1757 |
| 4 | P482.contig.3_2 | JHTS01000039.1 | 52533 to 58214 | BV82_3041 to BV82_3048 |
| 5 | P482.contig.3_2 | JHTS01000039.1 | 102734 to 107202 | BV82_3095 to BV82_3098 |
| 6 | P482.contig.3_2 | JHTS01000039.1 | 288172 to 296097 | BV82_3282 to BV82_3289 |
| 7 | P482.contig.3_8 | JHTS01000045.1 | 30598 to 54530 | BV82_3686 to BV82_3713 |
| 8 | P482.contig.3_11 | JHTS01000048.1 | 42147 to 47962 | BV82_4167 to BV82_4170 |
| 9 | P482.contig.3_11 | JHTS01000048.1 | 148657 to 158242 | BV82_4262 to BV82_4269 |
| 10 | P482.contig.3_30 | JHTS01000067.1 | 85537 to 95969 | BV82_5212 to BV82_5218 |
| 11 | P482.contig.3_30 | JHTS01000067.1 | 106848 to 115500 | BV82_5223 to BV82_5229 |
