# Supplementary material for: When Genome-Based Approach Meets the “Old but Good”: Revealing Genes Involved in the Antibacterial Activity of Pseudomonas sp. P482 against Soft Rot Pathogens
Source: Front Microbiol. 2016 May 26;7:782. doi: 10.3389/fmicb.2016.00782 (PMC4880745; doi:10.3389/fmicb.2016.00782)
Supplement: Supplementary file 9 [file Table9.DOCX]

Supplementary Material

**When genome-based approach meets the ‘old but good’: revealing genes involved in the antibacterial activity of *Pseudomonas* sp. P482 against soft rot pathogens**

Dorota M. Krzyżanowska^1^, Adam Ossowicki^1^, Magdalena Rajewska^1^, Tomasz Maciąg^1^, Magdalena Jabłońska^1^, Michał Obuchowski^2^, Stephan Heeb^3^, and Sylwia Jafra^1,*^

*** Correspondence:** Sylwia Jafra, [sylwia.jafra@biotech.ug.edu.pl](mailto:sylwia.jafra@biotech.ug.edu.pl)

**Supplementary Tables**

**Table S9.** Promoter regions and the transcription start sites predicted *in silico* for the studied part of cluster 18.

| **Sequence containing the promoter region (-10 and -35) and the transcription start site ^A^** | **Position in contig JHTS01000055.1^B^** | | **Strand** |
| --- | --- | --- | --- |
|  | **Start** | **End** |  |
| CCAGGTTTAACTCGCATTTAACAAAATTGTTAAATATCCG**A**TACCCAGAC | 30556 | 30605 | plus |
| TTCTTGCAATCTTCAGCCCCACAAATCAATAAAAGGACGT**T**CACGATGAG | 30871 | 30920 | plus |
| GTTGTCACTTTTAACAACGTTTCAATTTGTTTCTTATCCT**A**GGCAAGTTG | 30797 | 30846 | minus |

^A^ transcription start site is marked with the upper case letter

^B^ analyzed range of contig JHTS01000055.1: 27755 to 36623
