# Supplementary material for: When Genome-Based Approach Meets the “Old but Good”: Revealing Genes Involved in the Antibacterial Activity of Pseudomonas sp. P482 against Soft Rot Pathogens
Source: Front Microbiol. 2016 May 26;7:782. doi: 10.3389/fmicb.2016.00782 (PMC4880745; doi:10.3389/fmicb.2016.00782)
Supplement: Supplementary file 10 [file Table10.DOCX]

Supplementary Material

**When genome-based approach meets the ‘old but good’: revealing genes involved in the antibacterial activity of *Pseudomonas* sp. P482 against soft rot pathogens**

Dorota M. Krzyżanowska^1^, Adam Ossowicki^1^, Magdalena Rajewska^1^, Tomasz Maciąg^1^, Magdalena Jabłońska^1^, Michał Obuchowski^2^, Stephan Heeb^3^, and Sylwia Jafra^1,*^

*** Correspondence:** Sylwia Jafra, [sylwia.jafra@biotech.ug.edu.pl](mailto:sylwia.jafra@biotech.ug.edu.pl)

**Supplementary Tables**

# Table S10. Transcription terminator sequences predicted *in silico* for the studied part of cluster 18.

| **Transcription terminator sequence** | **Position in contig JHTS01000055.1^A^** | | **Strand** |
| --- | --- | --- | --- |
|  | **Start** | **End** |  |
| ACGTTGTTAAAAGTGACAACTACCCGCTCAACGATGT | 30827 | 30863 | plus |
| GGCGGGATTCTGCCTGCC | 32636 | 32653 | plus |

^A^ analyzed range of contig JHTS01000055.1: 27755 to 36623
