# Supplementary material for: When Genome-Based Approach Meets the “Old but Good”: Revealing Genes Involved in the Antibacterial Activity of Pseudomonas sp. P482 against Soft Rot Pathogens
Source: Front Microbiol. 2016 May 26;7:782. doi: 10.3389/fmicb.2016.00782 (PMC4880745; doi:10.3389/fmicb.2016.00782)
Supplement: Supplementary file 11 [file Image1.PDF]

Supplementary Material

When genome-based approach meets the 'old but good': revealing genes involved in the antibacterial activity of *Pseudomonas* sp. P482 against soft rot pathogens

Dorota M. Krzyżanowska[1], Adam Ossowicki[1], Magdalena Rajewska[1], Tomasz Maciąg[1], Magdalena Jabłońska[1], Michał Obuchowski[2], Stephan Heeb[3], and Sylwia Jafra[1,*]

*** Correspondence:** Sylwia Jafra, sylwia.jafra@biotech.ug.edu.pl

Supplementary Figure1

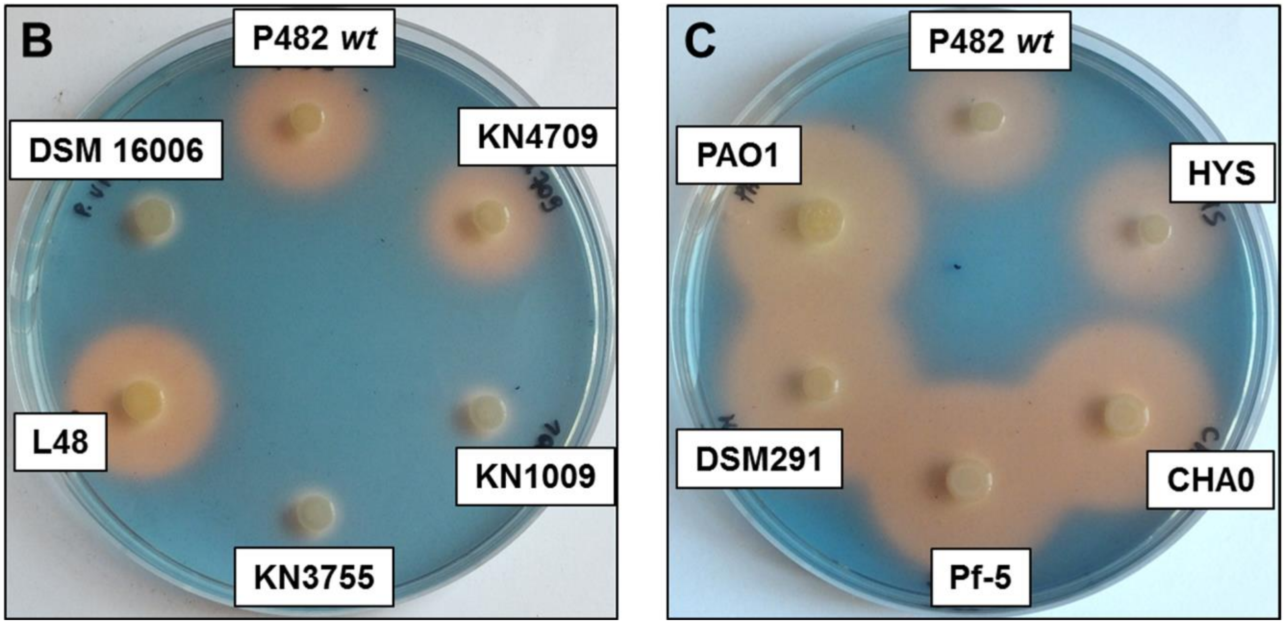

Fig. S1. Siderophore production assay on CAS blue agar. Panel A shows exemplary results for P482 and three mutants of this strain (Δ1009, Δ3755 and Δ4709). Panel B demonstrates a comparison of total siderophore production between *Pseudomonas* sp. P482 and seven other pseudomonads: *P. donghuensis* HYS^T, *P. protegens* CHA0^T, *P. protegens* Pf-5, *P. aeruginosa* PAO1, *P. putida* DSM291^T, *P. vranovensis* DSM 16006^T and *P. entomophila* L48 ^{T}. In our experimental setup, P482 and HYS^T produced a comparable amount of siderophores, yet markedly less than *Pseudomonas* spp.

strains CHA0^T, DSM 291^T and PAO1. This is in contrast with the results previously obtained for HYS^T, where this strain produced approx. 5-fold more siderophores than the mentioned pseudomonads (Gao et al., 2012; Yu et al., 2014).

Gao J, Yu X, Xie Z. Draft genome sequence of high-siderophore-yielding *Pseudomonas* sp. strain HYS. *J Bacteriol* (2012) 194**,** 4121-4121. doi: 10.1128/JB.00688-12.

Yu X, Chen M, Jiang Z, Hu Y,Xie Z. The two-component regulators GacS and GacA positively regulate a nonfluorescent siderophore through the gac/rsm signaling cascade in high-siderophore-yielding *Pseudomonas* sp. strain HYS. *J Bacteriol* (2014) 196**,** 3259-3270.
